# Protonation-dependent substrate release in a bacterial homolog of vesicular glutamate

**DOI:** 10.1016/j.bpj.2026.02.027

**Published:** 2026-02-21

**Authors:** Charles Plate, Natalia Dmitrieva, Samira Gholami, Mercedes Alfonso-Prieto, Sanket A. Deshmukh, Davide Mandelli, Paolo Carloni, Christoph Fahlke

**Affiliations:** 1 Department of Chemical Engineering, Virginia Tech, Blacksburg, Virginia; 2 Institute of Biological Information Processing (IBI-1) Molecular and Cell Physiology, Forschungszentrum Jülich, Jülich, Germany; 3 Institute of Neuroscience and Medicine (INM-9) Computational Biomedicine, Forschungszentrum Jülich, Jülich, Germany; 4 Department of Physics, RWTH Aachen University, Aachen, Germany

## Abstract

The SLC17 family contains diverse organic anion transporters with various stoichiometries and ion coupling mechanisms. A bacterial protein of this family, the D-galactonate transporter DgoT, co-transports two protons per substrate molecule. Although the overall transport cycle of DgoT has been proposed, the role of substrate protonation during its release remains unclear. Galactonate is expected to bind in a deprotonated form due to its low pKa; however, it can be released from the transporter in either a protonated or deprotonated state. In this study, we used well-tempered funnel metadynamics simulations to investigate the microscopic mechanisms underlying protonated and deprotonated galactonate dissociation from the inward-facing, gate-open conformation of DgoT. Our free energy profiles reveal that, although substrate protonation lowers the energy barrier for release and may enhance dissociation kinetics, deprotonated galactonate can also dissociate, albeit less frequently. These findings indicate that galactonate protonation facilitates, but is not strictly required for, substrate release by the bacterial organic anion transporter DgoT.

## Significance

The sugar-like molecule galactonate can be used by bacteria as a carbon source thanks to the D-galactonate transporter DgoT. Understanding its proton-coupled transport mechanism includes determining the protonation state of galactonate during substrate release to the cytoplasm. Simulations performed here show that galactonate is more easily released when it carries an extra proton, but it can still dissociate from the transporter without it. Thus, protonation of the titratable substrate emerges as an additional tactic for effective coupled transport.

## Introduction

Secondary active transporters utilize ion gradients to drive the movement of solutes across biological membranes, often operating with strict transport stoichiometries (1), which are ensured by the combination of the stoichiometric binding of several substrates and the controlled isomerization between inward- and outward-facing states (2). The SLC17 family of multifunctional secondary anion active transporters belong to the major facilitator superfamily (MFS) (3). It encompasses organic anion transporters that accumulate neurotransmitters in secretory vesicles (the vesicular glutamate (SLC17A6-8/VGLUT1-3) and nucleotide (SLC17A9/VNUT) transporters), remove carboxylated monosaccharides from lysosomes (SLC17A5/sialin), or extrude organic anions from the kidneys and the liver (SLC17A1-4/NPT1-4) (4). Diverse SLC17 transporters differ in transport coupling: VGLUTs function as H^+^-glutamate exchangers (5), sialin is an electroneutral H^+^-monosaccharide symporter (6), and VNUTs have been reported to function as ATP uniporters. Thus far, structural information has been obtained from VGLUT2 (7), sialin (8), and the bacterial model protein DgoT (9); the latter is the main D-galactonate (GAL) uptake carrier in *E. coli* (10), co-transporting two protons per one GAL. The three proteins feature 12 transmembrane (TM) helices that are organized into two symmetrical domains, each with six helices (see Fig. S1), which facilitate substrate binding and transport through an alternating-access mechanism (9,11).

We recently described the entire DgoT transport cycle with a combination of classical molecular dynamics (MD) simulations, Markov state modeling, and hybrid quantum mechanical/molecular mechanics MD simulations with experimental approaches (12). DgoT exhibits only two titratable residues in the transmembrane domain, D46 and E133 (Fig. S1); protonation of both acidic residues in the outward-facing conformation opens the extracellular gate and results in binding of galactonate (states 1–3 in Fig. S2). GAL is expected to bind in a deprotonated form because of its low pKa (3.39) in solution. After substrate association, the extracellular gate closes, facilitating transporter transition into an inward-facing occluded conformation (states 4–5 in Fig. S2). In this conformation, deprotonation of D46 (states 5–6 in Fig. S2) results in an open intracellular gate that permits GAL release. As D46 is sequestered from the intracellular solution but can rotate to approach E133, its deprotonation is likely to occur in a stepwise manner through E133 (Fig. S3). Initial proton release from E133, either to the intracellular solvent or to galactonate, is followed by H^+^ transfer from D46 to E133. After substrate dissociation, the second co-transported H^+^ is released from E133, resulting in intracellular gate closure and return of the empty transporter to an initial outward-facing conformation (states 7–8 in Fig. S2).

**Figure 1:**
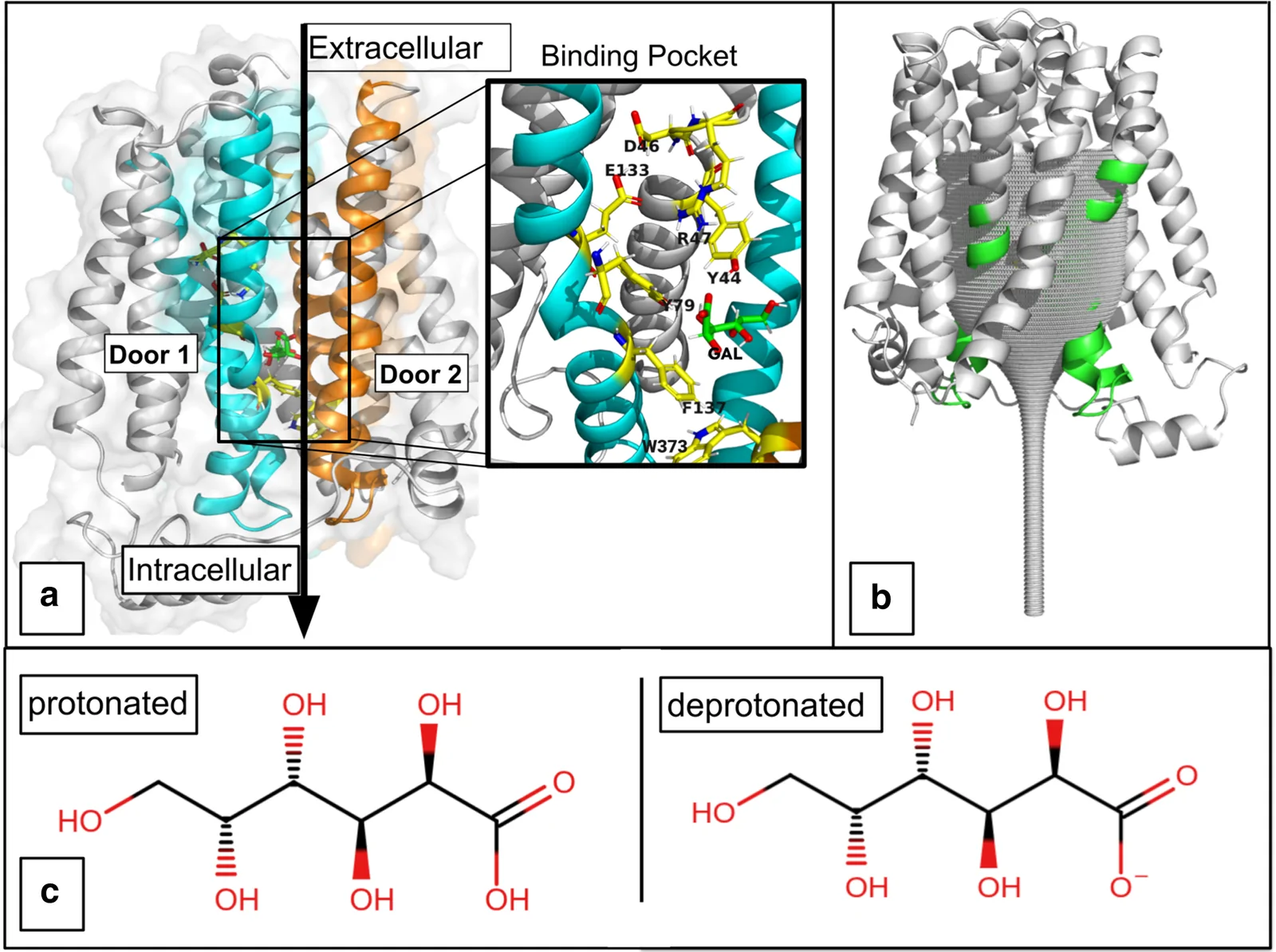
DgoT model used in our funnel metadynamics simulations, along with the structures of protonated and deprotonated galactonate. (*a*) Structure of DgoT (ribbon representation), with notable binding canal residues shown in yellow and the substrate (GAL) in green. The gating door 1 is formed by TM2 and TM3 in cyan, and door 2 is formed by TM8 and TM9 in orange, whereas TM11 was removed to ease visualization. (*b*) Funnel restraint volume overlaid on DgoT. Residues 139–158 (belonging to door 1) and residues 373–392 (door 2) are colored in lime and used to define the intracellular gate, which is restrained in an “open” state. (*c*) Structure of galactonate in its protonated and deprotonated states.

Our previous QM/MM simulations showed that the first released H^+^ can feasibly protonate GAL. However, spontaneous substrate release from the gate-open conformation in the D46[−]/E133[H] state (state 6 in Fig. S2) was observed for both deprotonated and protonated GAL in unbiased MD simulations (12). Therefore, it remains an open question whether GAL protonation is a crucial step in its dissociation. To obtain further insights into this key aspect, here, we performed enhanced sampling simulations. Specifically, we performed well-tempered funnel metadynamics (13,14) at a physiological temperature of 310.15K to investigate the diffusion of GAL in and out of the binding pocket (Fig. 1 *a*), using the funnel axis as our release coordinate (see supporting material methods for details). Moreover, to avoid closing of the intracellular gate upon substrate dissociation, we restrained it in its open position (Fig. 1 *b*). This choice is justified by extensive validation tests presented in the supporting material, where unbiased MD simulations show that the open intracellular gate is stable on the 500-ns timescale in the D46[-]/E133H state. By comparing the free energy profiles obtained with either deprotonated or protonated GAL (see Fig. 1 *c*), we uncovered kinetic and thermodynamic signatures that distinguish the more favorable substrate protonation state for spontaneous unbinding. Unbiased MD simulations, used to inform the construction of our metadynamics protocol, were consistent with the enhanced sampling results (see supporting material).

## Results and discussion

**Figure 2:**
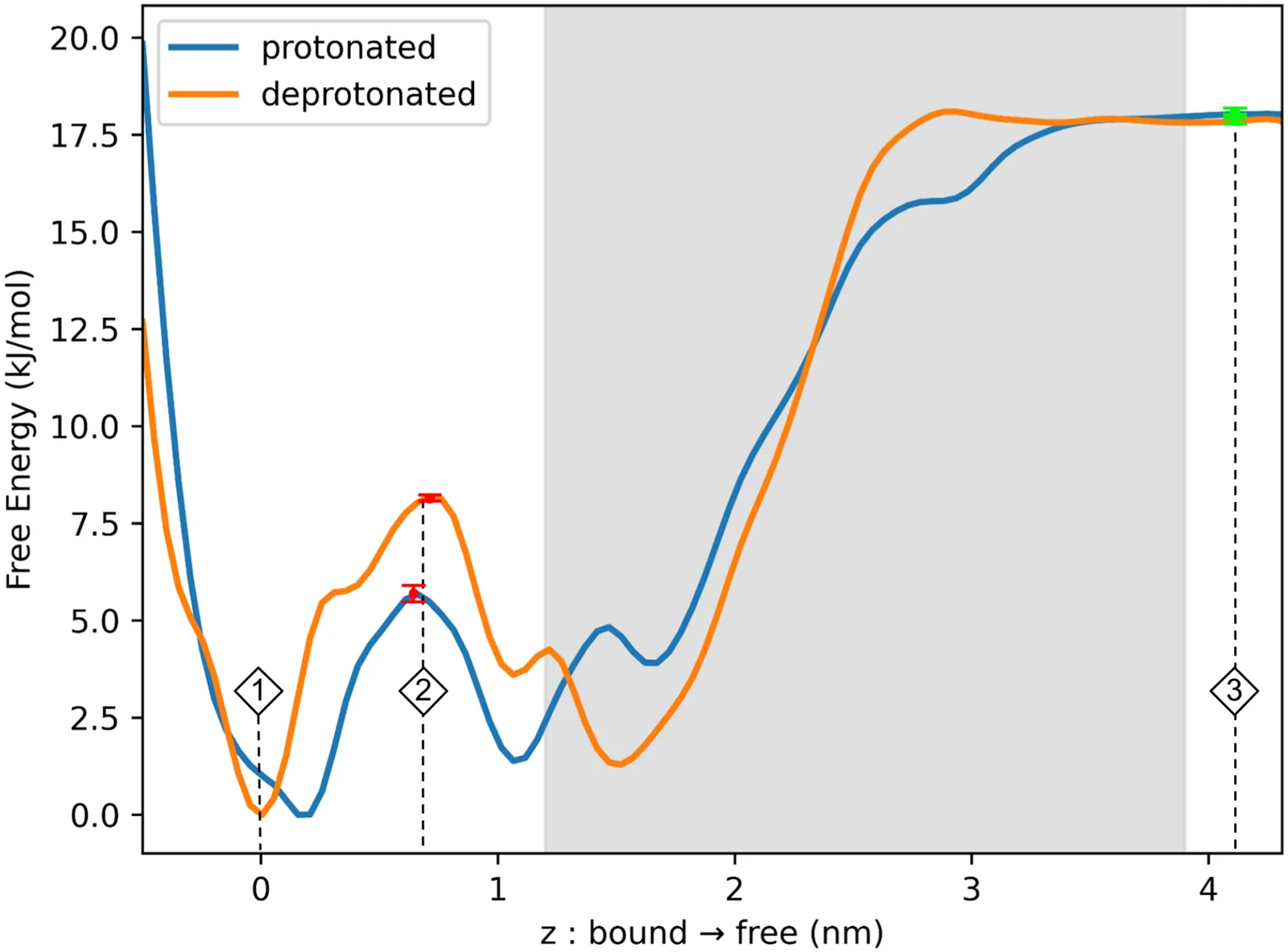
Free energy associated with the release of protonated (*blue*) and deprotonated (*orange*) GAL plotted as a function of its distance *z* from the binding pocket along the funnel axis. The shaded region represents the uncertain portion of the free energy: here, GAL interacts with both the funnel wall and the protein (see supporting materialmethods for details). Numbered diamonds denote characteristic points along the GAL release pathway: (1) bound (see structure in Fig. 3, *a* and *b*); (2) gate crossing barrier (see structure in Fig. 3, *c* and *d*); (3) fully solvated, unbound (see structure in Fig. S10). Error bars represent the standard deviations over the final 1000 ns of either metadynamics simulation for the (*red*) energy barrier from (1) to (2) and (*lime*) free energy of release from (1) to (3). The free energy of release extracted from the free energy profile must be corrected by applying the entropic funnel correction of −14.4 kJ/mol, as determined for our specific simulation setup (see Fig. S5).

**Figure 3:**
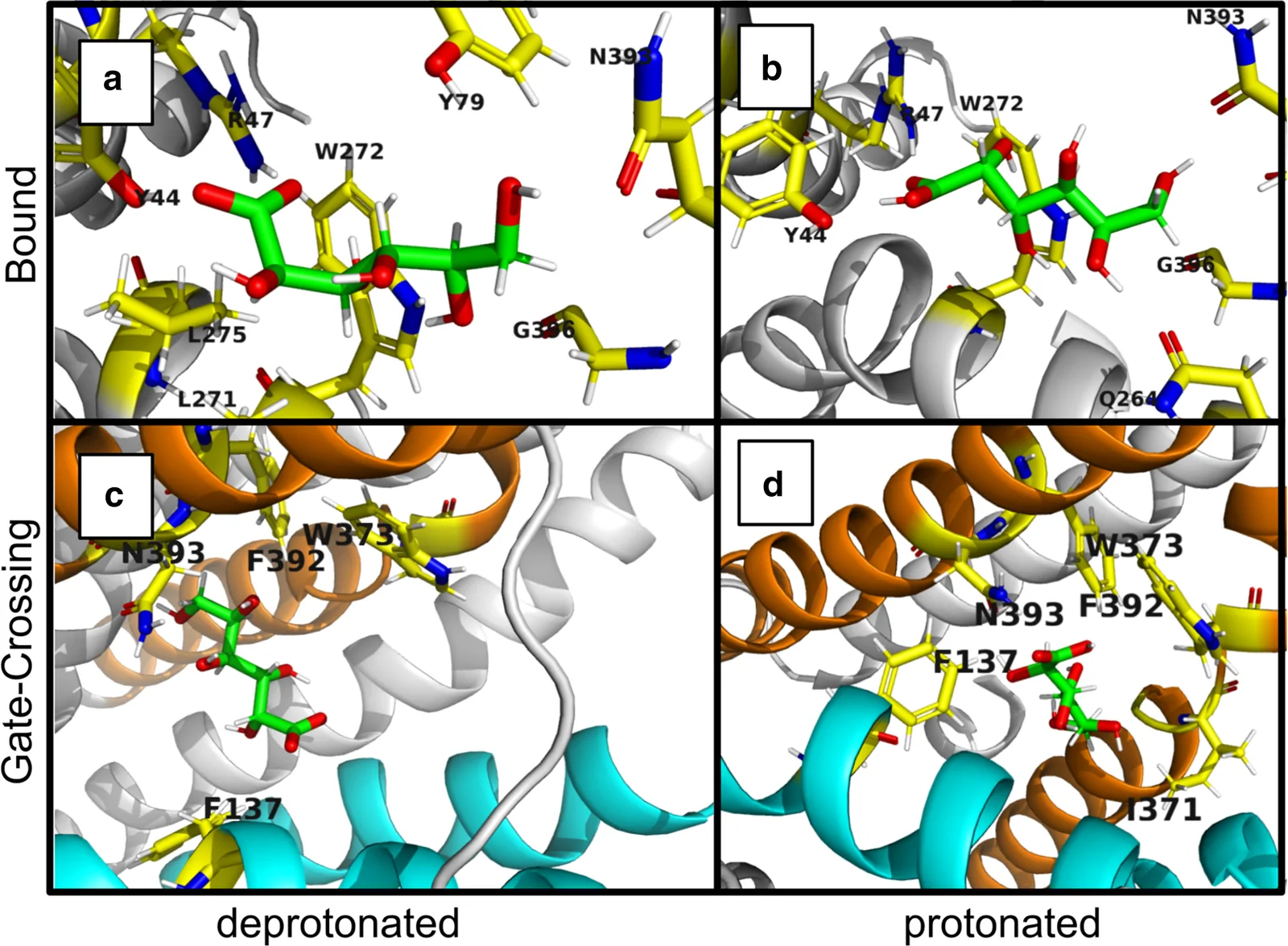
Representative structures of DgoT extracted from state 1 (bound) and state 2 (gate-crossing), as defined in Fig. 2. (a) Bound deprotonated galactonate. (b) Bound protonated galactonate. (c) Gate-crossing deprotonated galactonate. (d) Gate-crossing protonated galactonate. The protein frame is in gray, except door 1 and door 2 of the gate, which are colored cyan and orange, respectively. Residues exhibiting 3.5-Å contacts with galactonate are highlighted in yellow, and GAL is colored in lime. Snapshots for state 3 (unbound) in Fig. 2 are shown in the supporting material (see Fig. S10).

Fig. 2 reports the obtained 1D free energy profiles, with convergence tests shown in Figs. S4–S6. Energy barriers for release—defined as the highest energy point along the release pathway (marked 2) compared with the bound state (1)—are 8.2 ± 0.00 kJ/mol for deprotonated GAL and 5.7 ± 0.16 kJ/mol for protonated GAL. Although not staggering, the barrier height difference of 2.5 ± 0.16 kJ/mol between the two states suggests approximately 2.6 times faster release in the protonated state compared with the deprotonated state, according to Arrhenius kinetics. Evidence from unbiased MD simulations is consistent with the faster release for protonated galactonate, though the deprotonated form can also dissociate. Specifically, protonated galactonate was released in all four of four independent unbiased MD simulations within 500 ns, whereas release of the deprotonated substrate was observed in only two of four simulations of equal length (see Fig. S7). Moreover, the fully converged free energy of release, from the bound state (region around point 1; see also Fig. 3, *a* and *b*) to the unbound state (region around point 3; see also Fig. S10), for the protonated (3.4 ± 0.00 kJ/mol) and deprotonated state (3.0 ± 0.00 kJ/mol), turned out to be similar within the uncertainty of the simulations (<1%; see Fig. S5). Thus, the release process appears to be thermodynamically equivalent in the two protonation states. Such small free energy difference is consistent with both protomers being able to dissociate from the transporter, as observed in the unbiased classical MD simulations (Figs. S7–S9), as well as with the previous QM/MM MD simulations showing that the co-transported proton can be released either bound to galactonate or through water molecules (12). In contrast, the lower energy barrier observed for the protonated substrate points to protonation as a kinetic facilitator rather than a thermodynamic requirement. Overall, our results allow us to suggest that substrate protonation acts as a kinetic accelerator—affecting the rate but not the thermodynamic driving force—of substrate release in the inward-facing, open-gate conformation of DgoT. In other words, galactonate protonation provides kinetic control on substrate release without altering its thermodynamic driving force, thus allowing the transporter to ensure substrate dissociation regardless of its protonation state.

**Figure 4:**
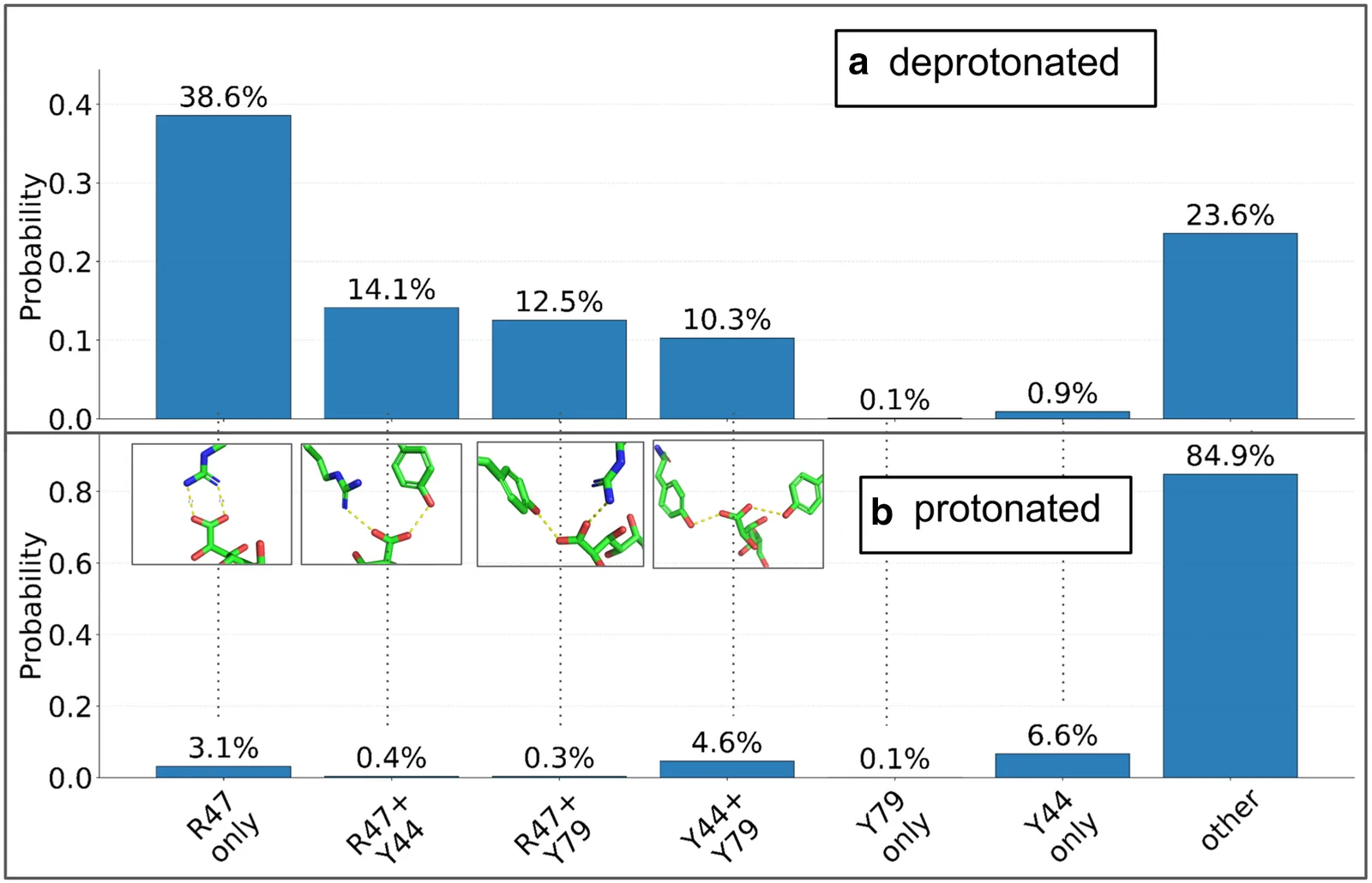
Percentage of bound frames exhibiting GAL-carboxyl interactions with specific residues, binned into discrete states. R47-only interaction comprises the first and second peaks of the R47-GAL (C-guanidinium to C-carboxyl) distance probability distribution representing the bidentate and monodentate contact modes, which extend to 5.0 Å (Fig. S14). Y44 and Y79 hydrogen bonds were classified based on the first peak of the GAL-Tyr (C-carboxyl to O-phenol) distance probability distribution up to 4.2 Å (Fig. S15). Panels (*a*) and (*b*) show the results of this analysis for deprotonated and protonated galactonate, respectively.

The difference in energy barriers can be largely attributed to deprotonated GAL being withheld in the transport canal by a salt bridge with R47 and ion-dipole interactions with the neighboring tyrosines Y44 and Y79 (Figs. 3 *a*, 4 *a*, and S11). Interaction with R47 in the bound state is more frequent (Figs. 4 and S11) and less flexible (Fig. S12, z coordinate below 0.5 nm) for the deprotonated galactonate form, resulting in the anionic substrate being less mobile in the binding site than the neutral form. Upon the substrate leaving the binding site, the behavior is inverted, with deprotonated galactonate showing larger fluctuations in the distance with R47. This allows us to suggest that R47 is the primary determinant of the bound-state stability for the anionic form, as well as the slightly higher barrier for deprotonated GAL. Attempting exit from these interactions, negatively charged GAL was immediately blocked by the bulky aromatic residue W373 that lines the intracellular gate (Fig. 3 *c*). Instead, the protonated (neutral) form of galactonate ceased to form comparable interactions with these crucial binding pocket residues (Figs. 3 *b*, 4 *b*, and S11). Although the contact between the hydrophobic W373 and the highly polar galactonate is unfavorable, it is less disadvantageous for the substrate neutral form (Fig. S13), resulting in overall smoother release for protonated galactonate. Our findings are consistent with the essential role of R47 in binding of the GAL carboxylic group evidenced by SSME experiments on the R47Q mutant (12).

The preferential release of protonated D-galactonate shown here and the regulation of intracellular gate opening by substrate protonation suggested by previous unbiased MD simulations (12) provide a possible explanation for a surprising set of recent mutagenesis results (12). In D46N DgoT, H^+^ transfer from E133 to protonate galactonate is still possible, but the resulting D46N/E133[-] state will exhibit a closed intracellular gate, in line with the observed lack of GAL transport. Instead, in E133Q DgoT, GAL protonation is precluded, but the intracellular gate can oscillate between closed and open states, clarifying why this mutant does not show net transport but still permits GAL exchange. Lastly, in D46N/E133Q DgoT, galactonate remains deprotonated, but a less tightly regulated intracellular gate opening will permit galactonate dissociation in either direction, explaining the observed GAL homoexchange.

During evolution, multiple mechanisms have developed to ensure strict stoichiometric transport in secondary active transporters. There exist stoichiometrically coupled binding, translocation between outward- to inward-facing conformations, or exclusive release at certain binding stoichiometry. In H^+^-coupled transporters, protonation of the titratable substrate as part of the transport cycle emerges as an additional tool for effective and coupled transport (15). Here we suggest that, although galactonate protonation is dispensable, it may enhance the kinetics of substrate release in the inward-facing, open-gate conformation of D46[-]/E133[H] DgoT.

Besides DgoT, SLC17 family encompasses other proton-coupled organic anion transporters (4), including the H^+^-glutamate exchangers VGLUTs (5) and the H^+^-sialic acid symporter sialin (8). The different proton coupling mechanisms across SLC17 members have been attributed to their different number and arrangement of protein titratable residues (Fig. S1). The results presented here and in (12) suggest that the organic anion substrate may also contribute to proton coupling, provided that it is titratable. VGLUTs transport glutamate into the acidic lumen of the synaptic vesicle, and the pKa values in bulk solution of galactonate (3.39) and glutamate (4.07–4.25) are within 1 pH unit, suggesting that the substrate could potentially be released in its protonated form also in VGLUTs. In contrast, substrate protonation is less likely in sialin, as sialic acid is more acidic (pKa = 2.60) than either glutamate or galactonate. Computational and experimental studies are needed to assess whether substrate protonation is a conserved strategy to facilitate substrate release across proton-coupled SLC17 transporters.

## Acknowledgments

C.P. gratefully acknowledges funding from the “Algorithms & Software for SUpercomputers with emerging aRchitEctures (ASSURE)” NSF-funded International Research Experiences for Students (IRES). D.M. and P.C. acknowledge support by the European Union’s HORIZON MSCA Doctoral Networks program, under Grant Agreement No. 101072344, project AQTIVATE (Advanced computing, QuanTum algorIthms and datadriVen Approaches for Science, Technology, and Engineering). This work was also supported by the 10.13039/501100001659Deutsche Forschungsgemeinschaft (German Research Foundation) to C.F. (FA 301/15–2), P.C. (CA 973/27-2), and M.A.-P. (AL 2511/1-2) as part of Research Unit FOR 2518, DynIon. The authors gratefully acknowledge the Advanced Research Computing (ARC) facility at Virginia Tech, and computing time on the supercomputer JURECA (16) at 10.13039/501100003163Forschungszentrum Jülich under grant no. dgot-gal.

## Declaration of interests

The authors declare no competing interests.

## Supporting citations

References (17,18,19,20,21,22,23,24,25,26) appear in the supporting material.

## Supporting material

Document S1. Figures S1–S18 and supplemental methods

Document S2. Article plus supporting material
