# Supplementary material for: Protonation-dependent substrate release in a bacterial homolog of vesicular glutamate
Source: Biophys J. 2026 Feb 21;125(7):1565–9. doi: 10.1016/j.bpj.2026.02.027 (PMC13351989; doi:10.1016/j.bpj.2026.02.027)
Supplement: Document S1. Figures S1–S18 and supplemental methods [file mmc1.pdf]

Biophysical Journal, Volume *125*

Supplemental information

Protonation-dependent substrate release in a bacterial homolog of vesicular glutamate

Charles Plate, Natalia Dmitrieva, Samira Gholami, Mercedes Alfonso-Prieto, Sanket A. Deshmukh, Davide Mandelli, Paolo Carloni, and Christoph Fahlke

Supporting Material for "Protonation-dependent substrate release in a bacterial homolog of vesicular glutamate transporters"

Charles Plate[1], Natalia Dmitrieva[2], Samira Gholami[2,3,†], Mercedes Alfonso-Prieto[3], Sanket A. Deshmukh[1], Davide Mandelli*[3], Paolo Carloni*[3,4], Christoph Fahlke[2]

[1]Department of Chemical Engineering, Virginia Tech, Blacksburg, VA 24061, USA
[2]Institute of Biological Information Processing (IBI-1) Molecular and Cell Physiology, Forschungszentrum Jülich, Wilhelm-Johnen-Straße, 52428 Jülich, Germany
[3]Institute of Neuroscience and Medicine (INM-9) Computational Biomedicine, Forschungszentrum Jülich, Wilhelm-Johnen-Straße, 52428 Jülich, Germany
[4]Department of Physics, RWTH Aachen University, 52056 Aachen, Germany

[†]Present address: Institute of Nanotechnology, Karlsruhe Institute of Technology, Kaiserstr. 12, 76131 Karlsruhe, Germany

*corresponding author

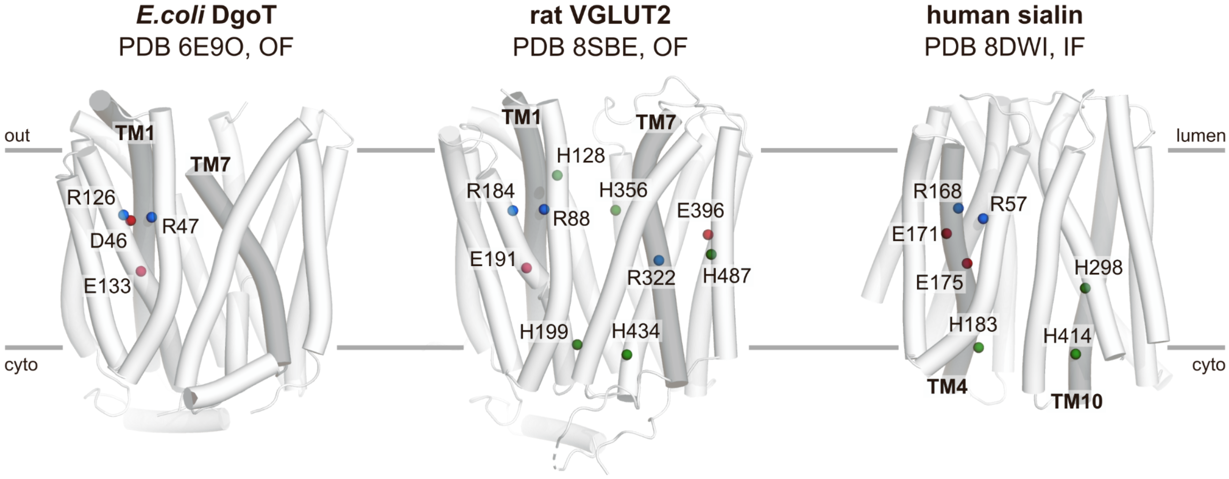

Figure S1. Comparison of DgoT with SLC17 transporters VGLUT2 and sialin. Representative experimental structures are shown with their Protein Data Bank (PDB) code and conformation (inward- or outward-facing, IF and OF, respectively). Gating helices are shown in darker gray, positions of titratable and charged amino acids in the transmembrane region of the respective proteins are shown in red (Asp/Glu), green (His) or blue (Arg/Lys). The horizontal line indicates the approximate location of the membrane and the SLC17 proteins are oriented so that the outside medium ('out) or the vesicular lumen ('lumen') is on top, whereas the cytoplasm is at the bottom.

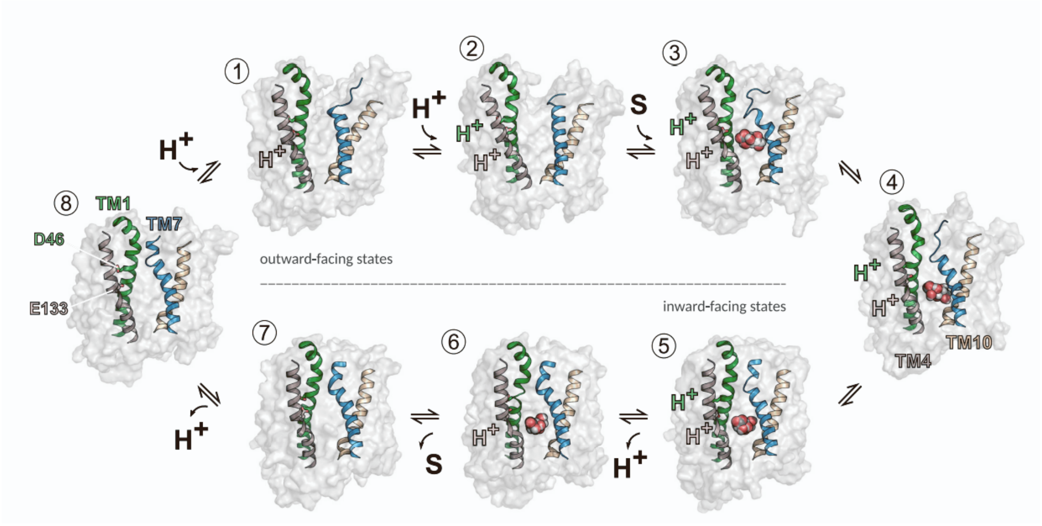

Figure S2: Transport cycle of DgoT (adapted from (1) under a Creative Commons CC-BY 4.0 license). The transport cycle of the bacterial transporter DgoT begins with the protein in an outward-facing conformation (1), where protonation of two acidic residues, D46 and E133, stabilizes the extracellular gate in an open state (2). This configuration allows the anionic substrate D-galactonate to bind from the extracellular side (3). Substrate binding induces conformational changes, leading to closure of the extracellular gate and transition into an occluded state where the substrate binding site becomes inaccessible from both sides of the membrane (4). Subsequently, DgoT undergoes a major conformational shift to an inward-facing state (5), enabling access to the cytoplasmic side. In this state, deprotonation of D46 opens the intracellular gate, permitting the release of galactonate, either in a protonated or deprotonated form (6). After substrate release, the intracellular gate closes, and deprotonation of E133 resets the transporter to its outward-facing conformation (7), ready for another transport cycle (8). In the present study we focus on substrate release from the inward-facing, open-gate conformation shown in (6).

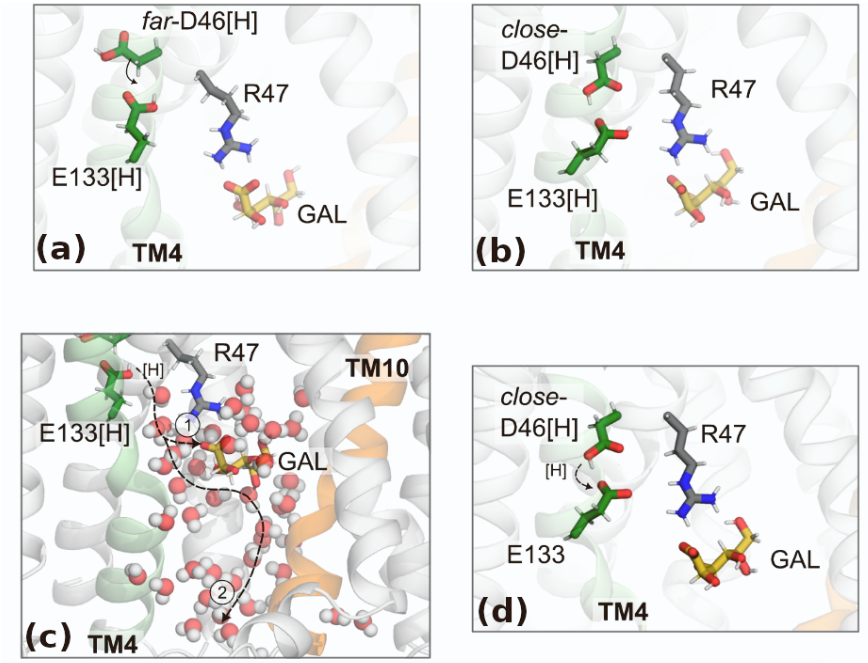

Figure S3: Schematic Illustration of the proton release in DgoT. (a) Initially, both D46 and E133 are protonated in IF DgoT, and side chain of D46 is in the *far* conformation. (b) After a conformational transition of D46[H] from a distant to a close conformation with respect E133 (*far*→*close*) a proton transfer pathway between D46 and E133 is formed. (c) Initial proton transfer from E133 to the nearby water molecule results in subsequent proton transfer either towards the substrate galactonate (pathway 1) or formation of a hydronium ion stabilized within the water network (pathway 2). Gating helices TM4 and TM10 are colored in green and orange, respectively, whereas TM2 and TM11 are hidden to reveal the permeation pathway. (d) Deprotonation of D46 facilitated by the now deprotonated E133, with the galatonate substrate still interacting with R47 before its release.

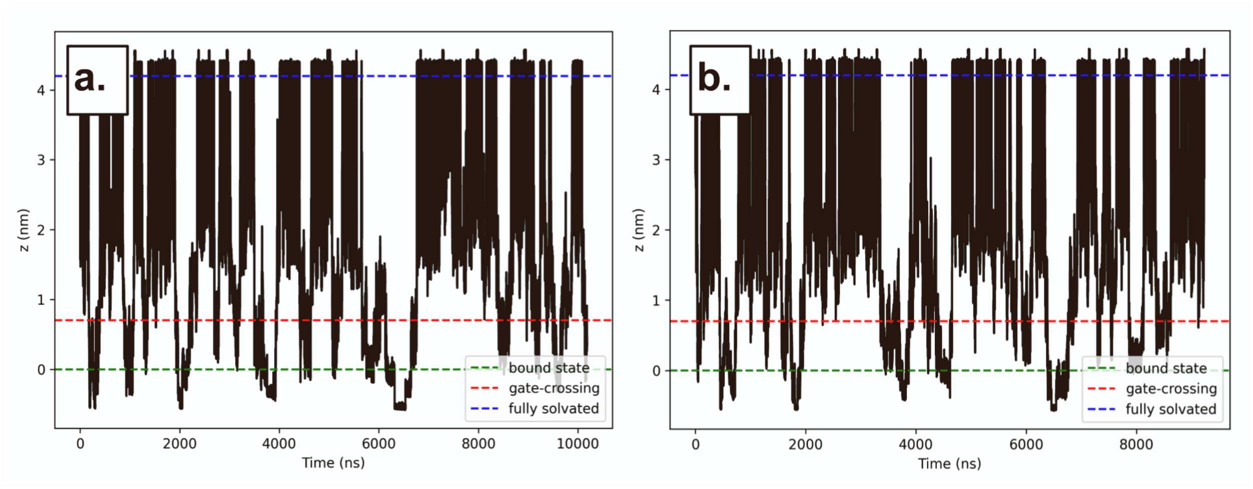

Figure S4: Time evolution of the *z* collective variable in the metadynamics simulation of the *deprotonated* (a) and *protonated* (b) system, showing many transitions between the bound and the unbound state of the substrate during ~10 μs.

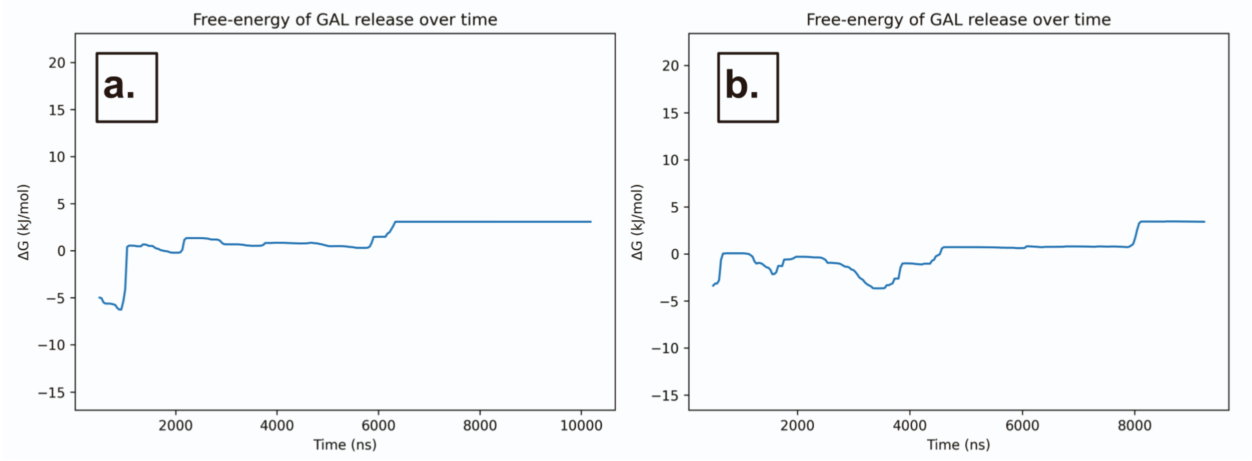

Figure S5: The figure reports the free energy of release, $\Delta G = -k_B T \log\left(\frac{\int_{\Delta_\mathrm{u}} e^{-\beta W(z)} \mathrm{d}z}{\int_{\Delta_\mathrm{b}} e^{-\beta W(z)} \mathrm{d}z}\right) + k_B T \log\left(C_0 \pi R^2 \Delta_u\right)$, between unbound (u) and bound (b) states as a function of time along the metadynamics trajectory, calculated using the instantaneous estimate of the 1D free energy surface W(z). The bound and unbound regions were defined as spanning the z-intervals Δ_b = (-0.1,0.1) nm, Δ_u = (4,4.2) nm for the deprotonated GAL and Δ_b = (0.1,0.3) nm, Δ_u = (4,4.2) nm for protonated GAL. The value of ΔG reported here already includes the entropic correction of $k_B T \log\left(C_0 \pi R^2 \Delta_u\right) =$ -14.4 kJ/mol, where R=0.1 nm is the radius of the cylindrical restraint in the unbound region, and C_0 is the standard concentration. The standard deviation calculated over the final 1000 ns of the trajectory corresponds to a relative error below 1% for both systems.

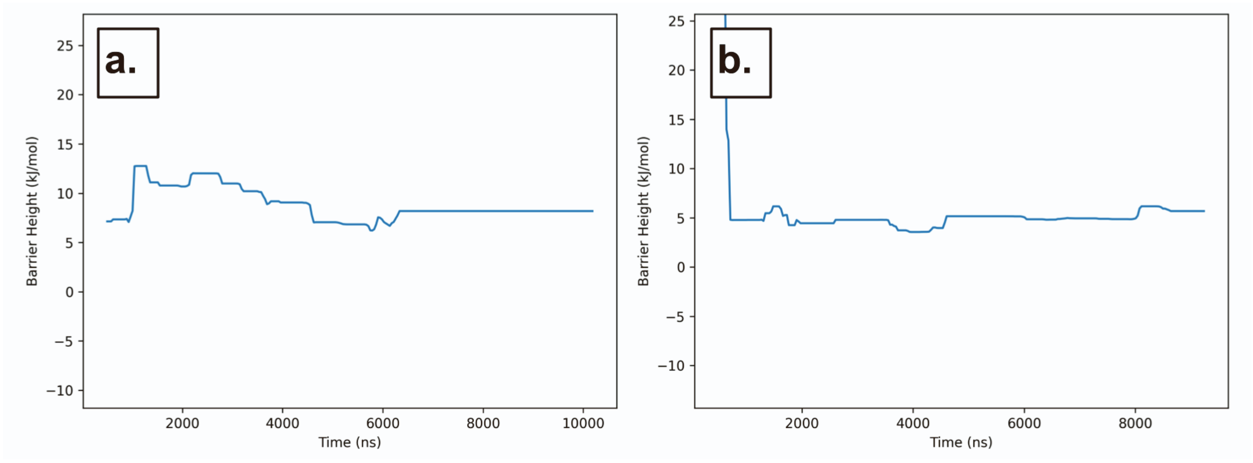

Figure S6: The energy barrier between bound and unbound states for deprotonated GAL (a) and protonated GAL (b), defined here as the free energy difference between the highest and lowest energy point on the directed path from z=-0.25 to z=1.2 nm. The standard deviation calculated over the final 1000 ns of the trajectory corresponds to a relative error of <1% for the deprotonated system and 2.7% for the protonated system.

			Replica / GAL Residence Time (ns)				
System	Protonation of GAL	Initial Gate Conformation	1	2	3	4	GAL Release
d-closed	[-]	closed	-1	-1	-1	-1	0/4
d-open	[-]	open	265	-1	39	-1	2/4
p-closed	[H]	closed	-1	-1	-1	-1	0/4
p-open	[H]	open	124	75	37	90	4/4

Figure S7. Residence time for galactonate release. A value of -1 indicates that no substrate release was observed within the duration of the simulation. We compared the release behavior of protonated and deprotonated galactonate in MD simulations of DgoT in its D46[-]/E133[H] protonation state. We considered four system configurations, controlling for the initial conformation of the intracellular gate: protonated (p) GAL with the intracellular gate either open or closed (*p-ope*n and *p-closed*, respectively*)* and deprotonated (d) GAL with the intracellular gate either open or closed (*d-ope*n and *d-closed*). For each system, we performed four independent 500 ns-long replica simulations, starting with the substrate in the binding site and recording its residence time, measured as the time before the ligand's center of mass exceeds z=3.9 nm. We found that while protonated GAL release occurred in all 4 gate-open replicas within the 500 ns trajectory length, deprotonated GAL dissociation was observed in only 2 out of 4. Notably, in simulations initiated with a closed gate (p-closed and d-closed), the intracellular gate remained stably closed in all four replicas. Likewise, simulations initiated with an open gate (p-open and d-open) remained predominantly open in all four replicas. This behavior suggests bimodality of gate conformation for the D46[-]/E133[H] protonation state, with an energy barrier larger than k_bT separating the open and closed states. Although the current sampling does not allow for a quantitative estimate of the substrate residence time, the results indicate that the protonation state of galactonate, while potentially facilitating release, is not a strict requirement for dissociation and release of both protonated and deprotonated GAL is feasible.

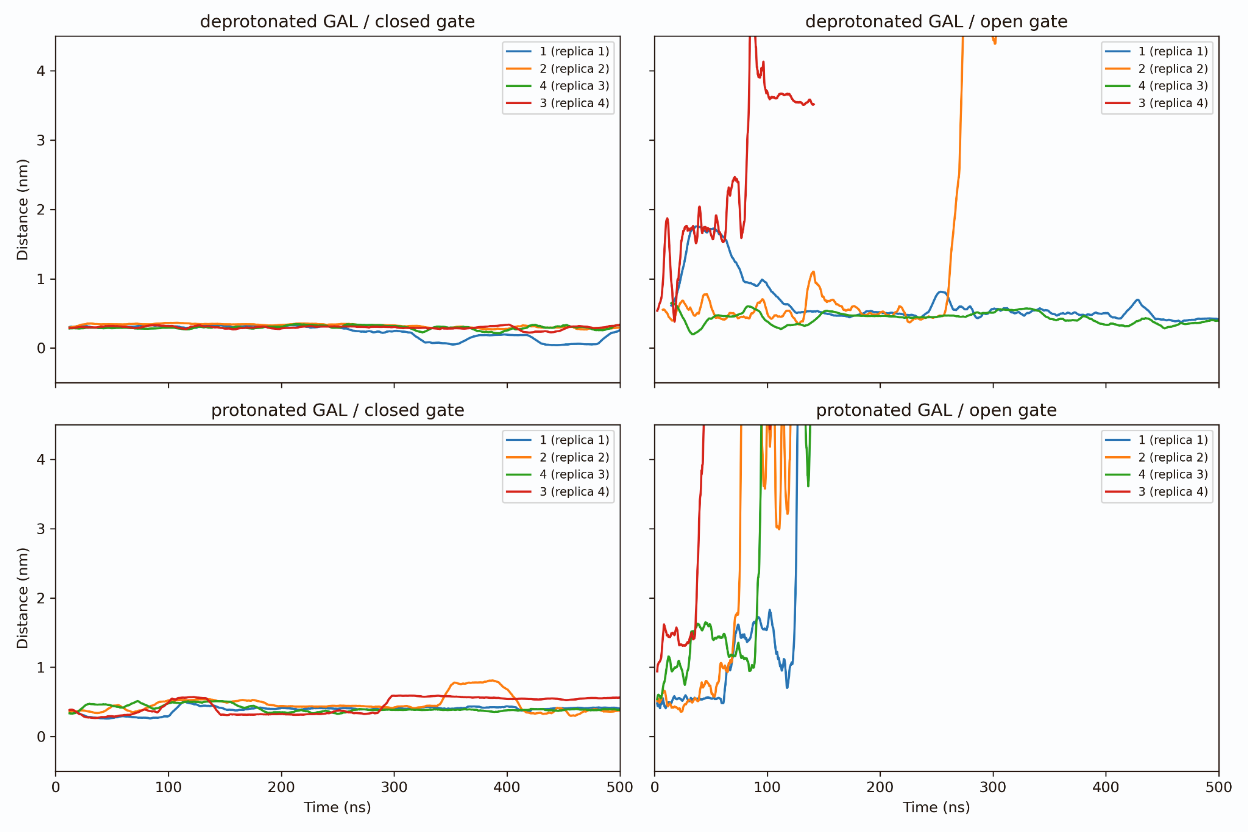

Figure S8: Distance from center of mass of protein to center of mass of galactonate in unbiased molecular dynamics simulations for the four systems investigated, as indicated in the titles of each panel.

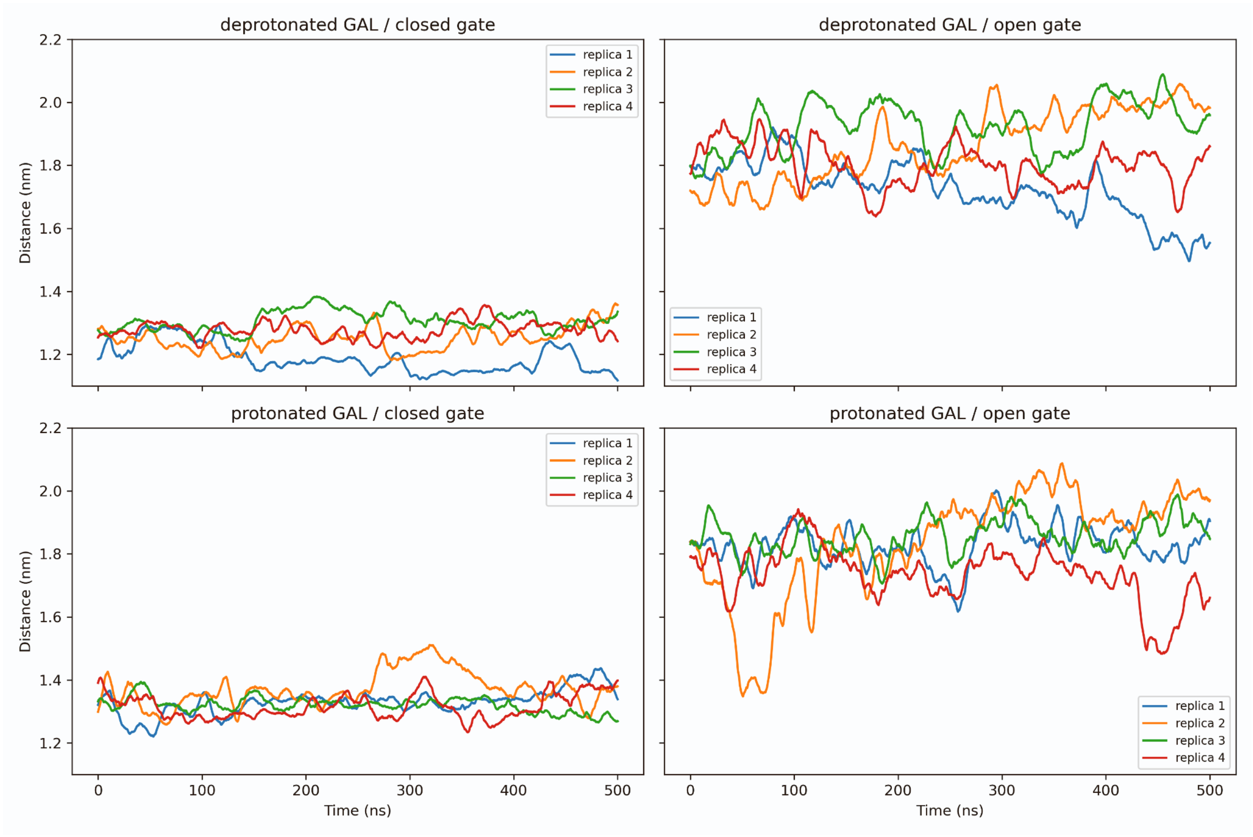

Figure S9: Gate distance during unbiased MD simulations, measured as a center of mass distance between the two “doors” described in the main text (see Fig. 1). Each panel refers to one of the four systems investigated, as indicated in the panel's title.

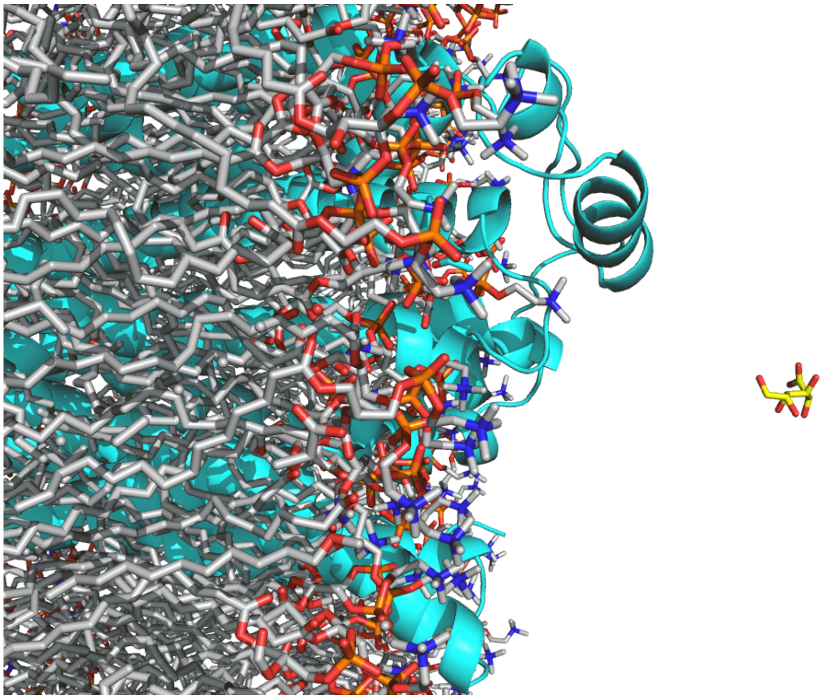

Figure S10: Galactonate Unbound state (point (3) at z=4.25 nm in Fig. 2 in the main text). Galactonate (yellow-carbon), DgoT (cyan cartoon), Lipid Bilayer (gray-carbon).

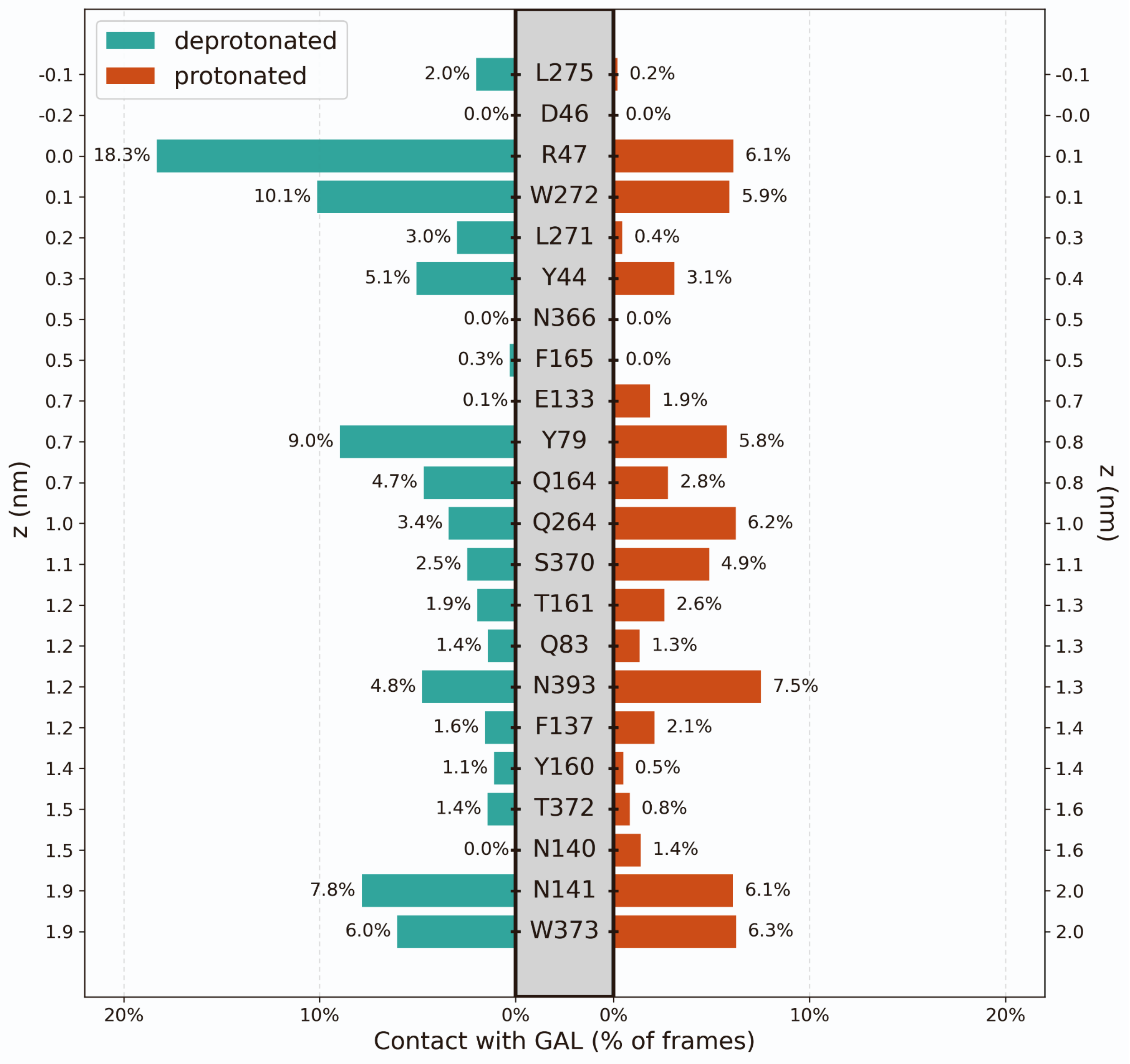

Figure S11: Binding pocket residue contacts with galactonate, using a 3.2 Å all-atom minimum distance. Left and right y-axes portray the average z-coordinate of the center-of-mass of residues oriented on the chart from binding pocket (top) to gate (bottom) during the simulations of deprotonated and protonated GAL.

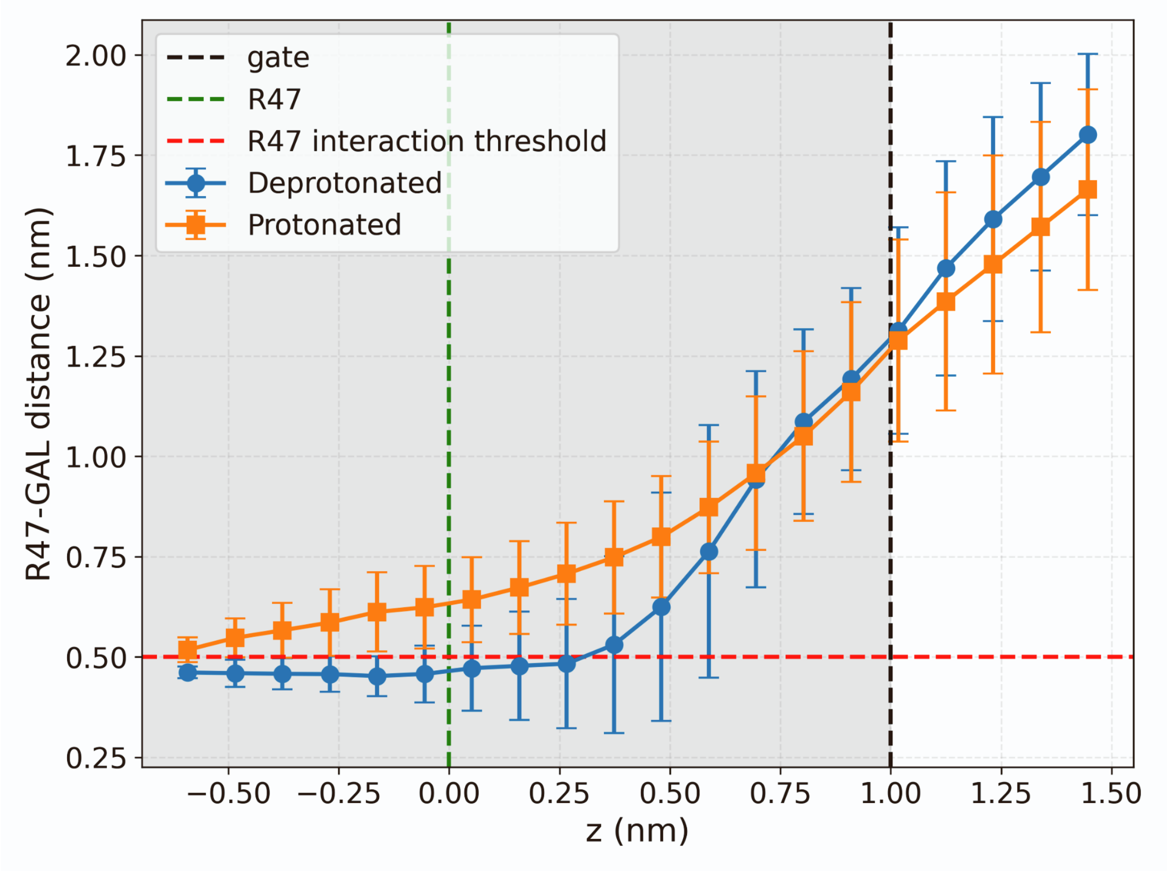

Figure S12. Average R47 C-guanidinium to GAL C-carboxyl with standard deviation, as a function of the reaction coordinate z. Distance values were binned with 0.1 nm binwidth and each mark represents the center of a bin. The black dashed line represents the location of the intracellular gate, whereas the green dashed line shows the location of the R47 residue. The red dashed line indicates an approximate threshold for interaction with R47 (0.50 nm), above which the interaction can be considered weak or broken. The shaded portion of the graph represents the bound state region (i.e. before the intracellular gate).

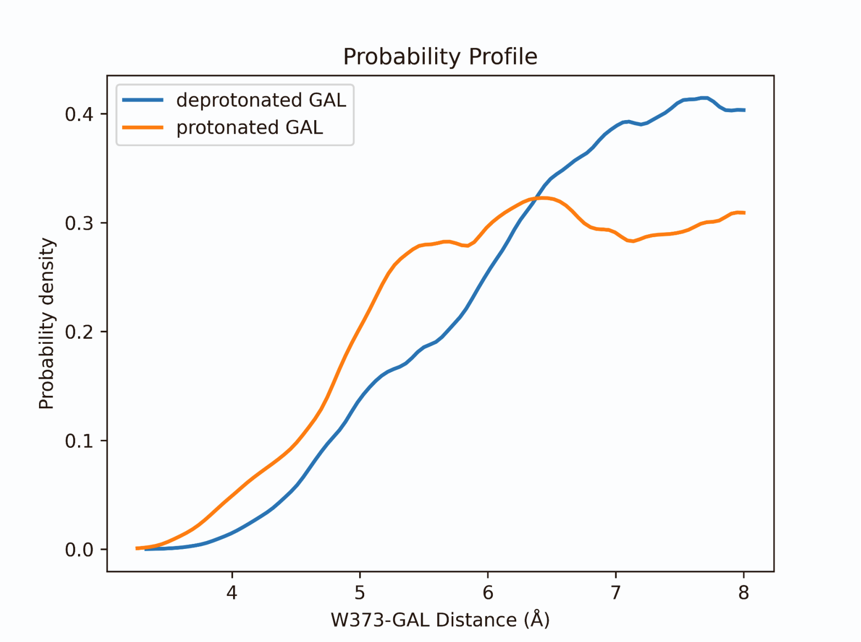

Figure S13: Distance between the center of mass of W373 and the carboxyl carbon of galactonate.

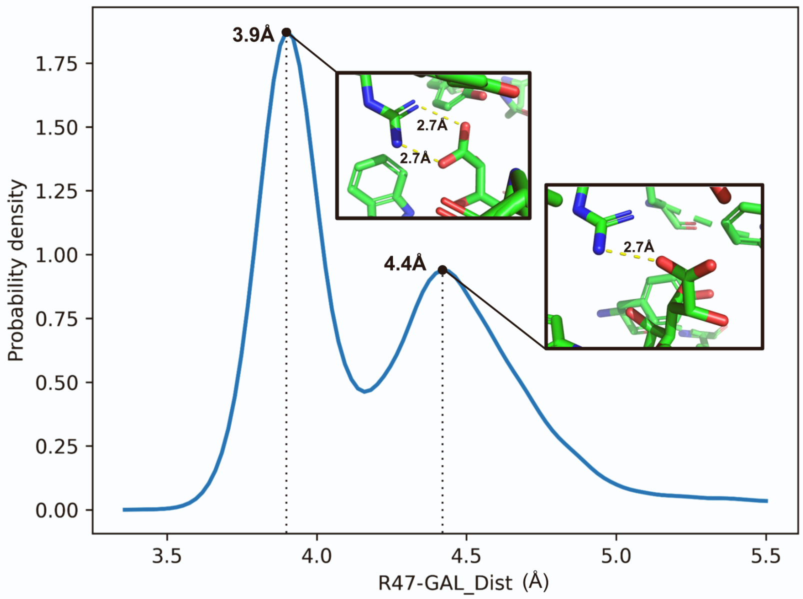

Figure S14: Distance probability distribution measuring the distance *R47 (C-guanidinium*) to *GAL (C-carboxyl)* for the *deprotonated* galactonate simulation. First and second peaks at 3.9 and 4.4 Å show bidentate and monodentate contacts, respectively.

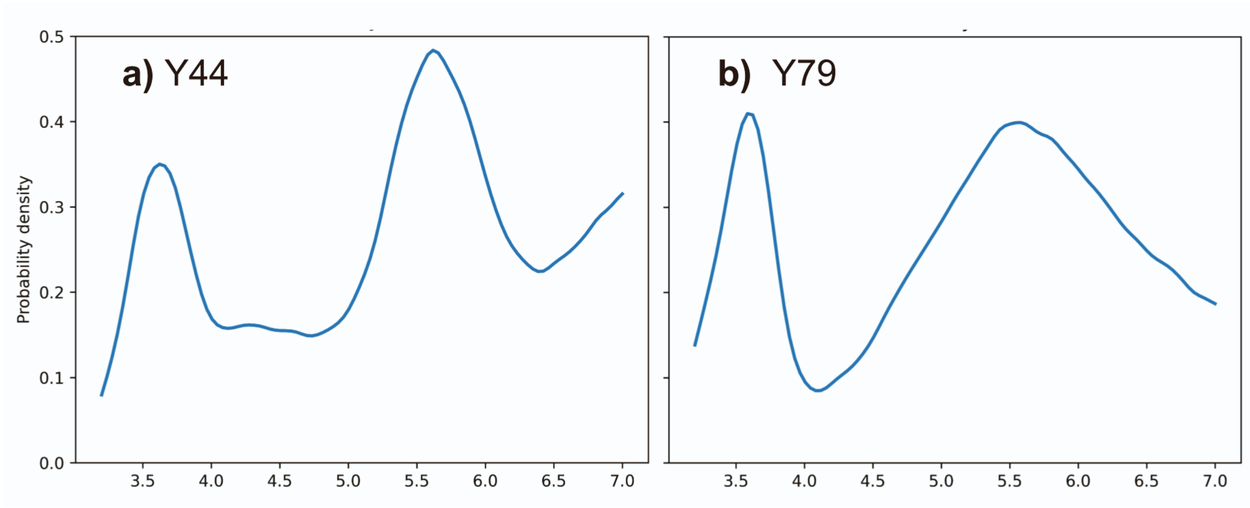

Figure S15: Distance probability distribution between GAL *(C-carboxyl)* and a) Y44 or b) Y79 (C-carboxyl – O-phenol) for the *deprotonated* galactonate simulation. The first peak until 4.2 Å represents a hydrogen bond between GAL and tyrosine.

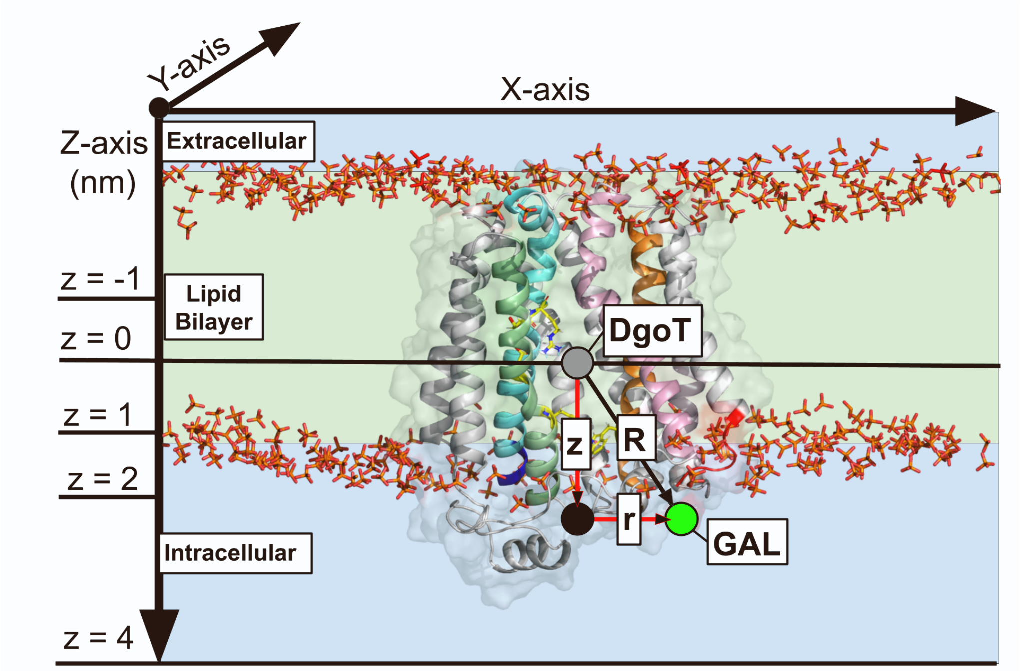

Figure S16: System coordinates: the displacement vector ***R*** is the center of mass of GAL minus the center of mass of DgoT*;* ***z*** is simply the z-component of *R* (R.z) and corresponds roughly to the release coordinate of GAL; ***r*** is the radius used in the funnel restraint calculated as $\sqrt{R.x^2 + R.y^2}$

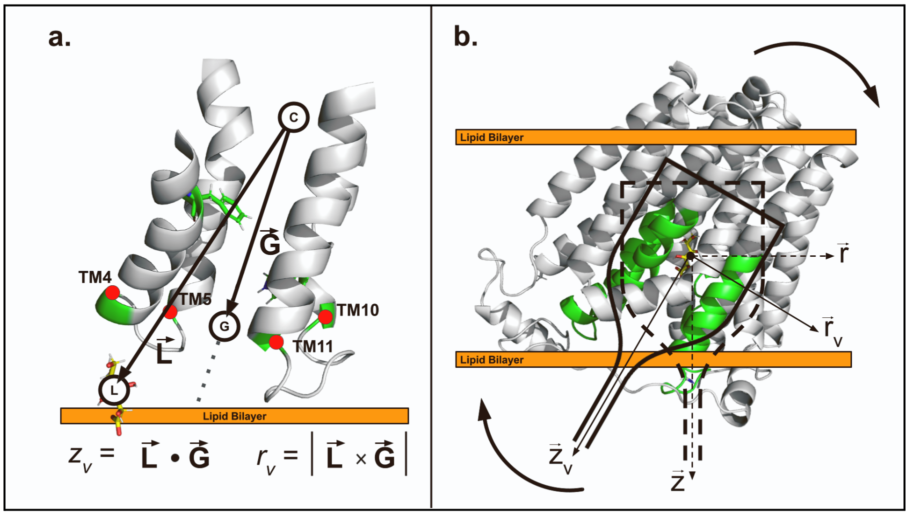

Figure S17: Rotationally invariant collective variables. The vector $\vec{G}$ in panel A is a unit vector directed from the center of mass of the protein *C* to point *G* which denotes the center of the 4 Cα atoms located at the ends of the gating helices TM4 and TM10 (containing the intracellular gate residues F137 and W373 shown as lime sticks), as well as the adjacent helices TM5 and TM11, denoted with red points in the right panel. $\vec{L}$ points from *C* to the center of mass of the ligand *L*. Our final invariant GAL release coordinate z_v (see panel B) is thus given by $\vec{L} \bullet \vec{G}$ and the invariant radius r_v which is necessary to construct the walls of the funnel, is computed as $|\vec{L}$ x $\vec{G}|$.

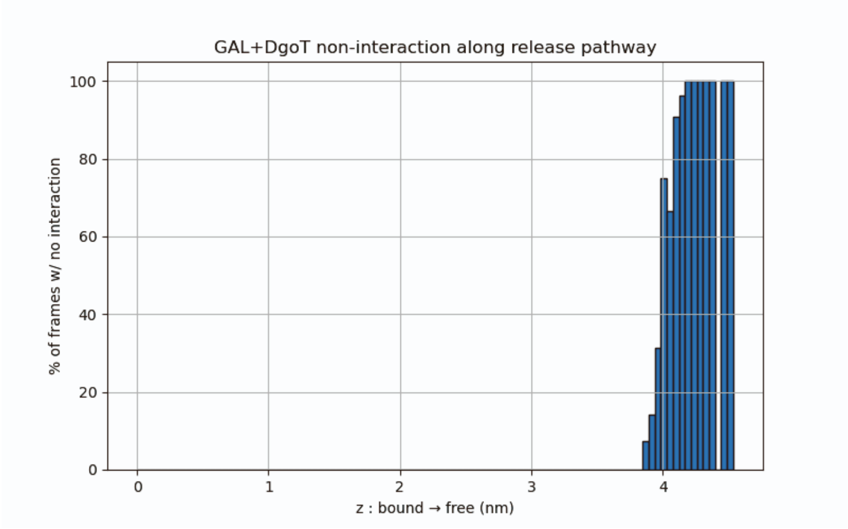

Figure S18: Interaction between DgoT and GAL substrate along the release reaction coordinate. Bars show the percentage of frames with minimum distance between atoms of DgoT and atoms of GAL exceeding 12 Å – the Lennard Jones and Coulomb interaction cutoff of the simulation.

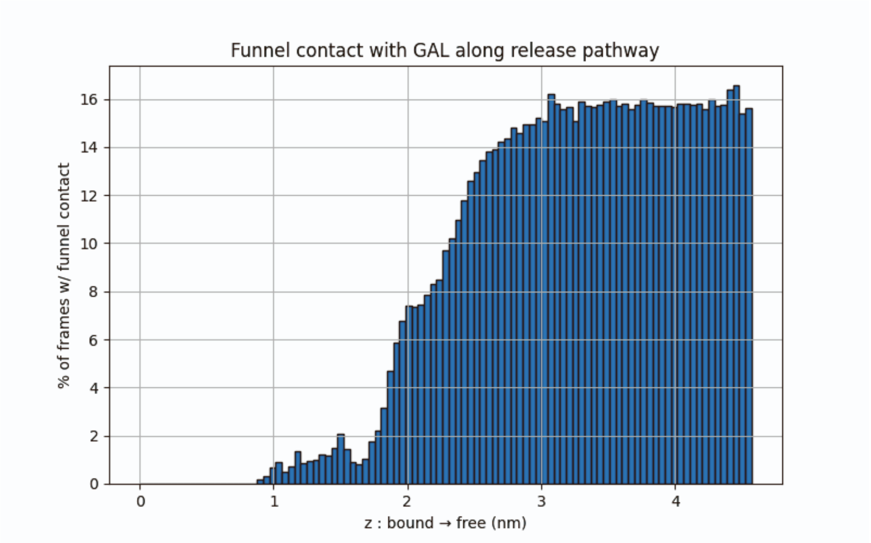

Figure S19: Interaction between GAL substrate and funnel walls along the release reaction coordinate. Bars show the percentage of frames where the restraint force from the funnel wall was not equal to 0.

Methods

MD Simulation Details. The setup of the simulations follows our previous work (1). Standard protonation states at neutral pH were assigned to all protein titratable residues (deprotonated aspartate and glutamate residues, and singly protonated histidine residues, except for H56, which forms a salt bridge with E180 and, therefore, was set as doubly protonated). The proteins were embedded in a POPC bilayer and surrounded by a 100mM NaCl solution. The overall systems were neutral. The CHARMM36m force field (3,4) was used for the protein and lipids. Ions were described using default CHARMM parameters, and the CHARMM TIP3P model (5) was used for water molecules. Protonated and deprotonated galactonate (GAL) parameters were taken from (1). They were obtained using the SwissParam server (6) in both protonated and deprotonated states. Van der Waals interactions were calculated with the Lennard–Jones potential and a cutoff radius of 1.2 nm, with forces smoothly switched to zero in the range of 1.0–1.2 nm. Electrostatic interactions were calculated by the particle mesh Ewald method (7), with a real-space cutoff distance of 1.2 nm. An integration time step of 2 fs was used. In all simulations the temperature was maintained at 310.15 K using the v-rescale thermostat (8) with a time constant of 0.5 ps. The thermostat was applied separately to the protein, lipid bilayer, and aqueous solution containing ions. The same groups were used for the removal of the center-of-mass linear motion. The systems were first equilibrated following the protocol described in (1). After that, 500 ns-long production MD runs for each of the two galactonate protomers were performed in the isothermal-isobaric ensemble using a semi-isotropic Parrinello–Rahman (9) barostat with a time constant of 0.5 ps, at the target pressure to 1 bar. The last configuration obtained from these production runs were used as the starting points for subsequent ~10 μs-long well-tempered funnel metadynamics simulations.

Well-Tempered Funnel Metadynamics Simulations. Funnel metadynamics was used to drive the galactonate unbinding process. The release coordinate (z) – also describing the axis of the funnel, was defined as the vector pointing from the center of mass of the protein through the mouth of the gating helices (Fig. S16-S17). We restrained the center-of-mass of GAL within a funnel in the shape defined by the sigmoid function

$$r(z) = R_w + \frac{R_t - R_w}{1+\exp[-4m(z-C)/(R_w-R_t)]}$$

which decays smoothly from a wide radius (R_w) to a thin radius (R_t). The inflection point (C) of the curve defines the midpoint of this decay, while the slope (m) sets the steepness of the drop-off. We used C=1.5 nm, m=1 nm, R_w=1.2 nm, and R_w=1 Å. The latter was chosen following (2). The resulting funnel restraint volume extends from 0.5 nm above the protein center-of-mass to 4.25 nm beyond and into the solvent.

The gate, which was restrained in the open conformation during metadynamics, was defined as the center of mass distance between door-1 (spanning residues 139-158) and door-2 (spanning residues 373-392). A lower bound on this distance was enforced at 1.7 nm, at which point a harmonic restraint with a hard energy constant of 25,000 kJ mol^{-1} would kick in. To stop the secondary structure in the doors from warping under the gate restraint force, a dihedral restraint

with a force constant of 1,000 kJ mol^{-1} nm^{-1} was applied to the backbone atoms of the residues in the doors.

Well-tempered metadynamics simulations (10) were performed using the PLUMED library version 2.6.2 (11) plugged into GROMACS version 2024.3 (12). Gaussian potentials with a height of 1.2 kJ/mol and a width (σ) of 0.1 nm were deposited every 500 simulation steps. A bias factor of 25 was applied to modulate the bias deposition and ensure convergence of the free-energy surface.

In order to affirm the reliability of our free energy estimates, it was necessary to mark regions of the reaction coordinate (*z*) as valid or invalid (shaded gray in Fig. 3 of the main text) and ensure that any numerical claims pointed to valid regions of the free energy surface. Namely, a region of the free energy surface was deemed invalid if GAL was feeling a restraint force from the funnel walls in >1% of frames, while interacting with DgoT in >1% of frames. In the protein bound region from z = (-0.25 to 1.2) nm, GAL was interacting with DgoT but not experiencing any bias from the funnel restraint (see Fig. S18-S19). In the transition region from z=(1.2 to 3.9) nm where the funnel narrows, GAL frequently interacts with DgoT, but the restraint force from the funnel walls biases its interactions with the protein, clouding the reliability of the surface (see Fig. S18-S19). Finally, in the solvated state z=(3.9 - 4.2) nm, the ligand is beyond the 12 Å interaction cutoff distance and is thus confirmed to experience no force from the protein (Fig. S18-S19). At this point, GAL is restrained to a homogeneous volume outside the protein, allowing for a proper volume correction to be applied, and the free energy of release to be determined, as the difference between bound and unbound states.

Release of Galactonate from Unbiased MD Simulations. We focused on the state that features deprotonated D46 (or D46[-]) and protonated E133 (E133[H]), as deprotonation of D46 was found to be crucial for substrate release in our previous classical MD simulations (1). For GAL, two protomers were considered, protonated (*p* hereafter) and deprotonated (*d*) (see Fig. 1c of the main text). The starting structures of the corresponding DgoT/GAL complexes in their inward-facing state were taken from the trajectories obtained in our previous work (1) (denoted hereafter as *root-p* and *root-d*, respectively for protonated and deprotonated GAL). Specifically, from the *root-p* simulation*,* we selected one snapshot in which the gate was opened (defined if the F137/W373 distance is greater than 8 Å) to obtain the protonated GAL and gate opened state (*p-open* system). Next, we deprotonated the GAL molecule from the latter (and remove one chloride ion to maintain neutrality). The resulting system was optimized using a steepest descent algorithm with a maximum force tolerance of 10 kJ mol^{-1} nm^{-1} on each atom. This led to the *d-open* system, with deprotonated GAL and the gate open. Complementarily, we selected a snapshot from the *root-d* simulation with the gate closed (defined if the F137/W373 distance is lower than 8 Å) to obtain the *d-closed* system. From this configuration, we protonated the GAL (and removed one sodium ion) to obtain the *p-closed* system, which was then minimized using the same protocol as for the *d-open* system. Therefore, we generated a total of four initial structures, controlling for both the protonation state of GAL and the open/closed state of the gate. These were used as starting structures for unbiased MD simulations. Specifically, for each of the four distinct states (*d-open, d-closed, p-open, p-close*) we performed four distinct 500 ns NPT runs (i.e., 16 simulations or 8 μs in total), where the initial velocities were extracted from a Maxwell-Boltzmann distribution at the target temperature of 310.15 K. These simulations were used to investigate the time for release of the substrate. This was implemented by monitoring the distance from the center of mass of GAL to that of the protein projected onto the intracellular-bound axis perpendicular to the bilayer. The simulation was stopped if this metric exceeded the z=3 nm mark, corresponding to the cytosol-membrane interface (Fig. S10). This procedure yielded the estimates of the residence time listed in Fig. S7.

Validation of the Open Gate Restraint:

The results of the unbiased MD simulations also provide important validation for our choice of restraining the intracellular gate open during the metadynamics simulations. The gate remained open in trajectories initiated with an open conformation and those started from closed conformations showed a closed gate for 500 ns (Figs. S7-S9). This indicates that the gate conformation is stable on this timescale and that its open and closed states are separated by an energy barrier larger than k_BT. Spontaneous substrate release was observed only in unbiased MD simulations initiated from the open-gate conformation, whereas no release occurred when the gate was closed, suggesting that gate opening is a prerequisite for release. Furthermore, no spontaneous gate closure was observed following substrate release in the 500 ns timescale of the unbiased MD simulations. This is in line with the results of ref.(12), showing that (i) deprotonation of D46 favors the open state of the intracellular gate and (ii) subsequent release of the second proton is necessary for intracellular gate closure upon substrate dissociation.

Supporting References

1. Dmitrieva, N., Gholami, S., Alleva, C., Carloni, P., Alfonso-Prieto, M., & Fahlke, C. (2024). Transport mechanism of DgoT, a bacterial homolog of SLC17 organic anion transporters. The EMBO Journal, 43(24), 6740–6765. https://doi.org/10.1038/s44318-024-00279-y
2. Raniolo, S., & Limongelli, V. (2020). Ligand binding free-energy calculations with funnel metadynamics. Nature Protocols, 15(9), 2837–2866. https://doi.org/10.1038/s41596-020-0342-4
3. Klauda, J. B., Venable, R. M., Freites, J. A., O'Connor, J. W., Tobias, D. J., Mondragon-Ramirez, C., Vorobyov, I., MacKerell, A. D., & Pastor, R. W. (2010). Update of the CHARMM All-Atom Additive Force Field for Lipids: Validation on Six Lipid Types. The Journal of Physical Chemistry B, 114(23), 7830–7843. https://doi.org/10.1021/jp101759q
4. Huang, J., Rauscher, S., Nawrocki, G., Ran, T., Feig, M., de Groot, B. L., Grubmüller, H., & MacKerell, A. D. (2016). CHARMM36m: an improved force field for folded and intrinsically disordered proteins. Nature Methods, 14(1), 71–73. https://doi.org/10.1038/nmeth.4067
5. Jorgensen, W. L., Chandrasekhar, J., Madura, J. D., Impey, R. W., & Klein, M. L. (1983). Comparison of simple potential functions for simulating liquid water. The Journal of Chemical Physics, 79(2), 926–935. https://doi.org/10.1063/1.445869
6. Zoete, V., Cuendet, M. A., Grosdidier, A., & Michielin, O. (2011). SwissParam: A fast force field generation tool for small organic molecules. Journal of Computational Chemistry, 32(11), 2359–2368. Portico. https://doi.org/10.1002/jcc.21816
7. Essmann, U., Perera, L., Berkowitz, M. L., Darden, T., Lee, H., & Pedersen, L. G. (1995). A smooth particle mesh Ewald method. The Journal of Chemical Physics, 103(19), 8577–8593. https://doi.org/10.1063/1.470117
8. Bussi, G., Donadio, D., & Parrinello, M. (2007). Canonical sampling through velocity rescaling. The Journal of Chemical Physics, 126(1). https://doi.org/10.1063/1.2408420
9. Parrinello, M., & Rahman, A. (1981). Polymorphic transitions in single crystals: A new molecular dynamics method. Journal of Applied Physics, 52(12), 7182–7190. https://doi.org/10.1063/1.328693
10. Barducci, A., Bussi, G., & Parrinello, M. (2008). Well-Tempered Metadynamics: A Smoothly Converging and Tunable Free-Energy Method. Physical Review Letters, 100(2). https://doi.org/10.1103/physrevlett.100.020603
11. Tribello, G. A., Bonomi, M., Branduardi, D., Camilloni, C., & Bussi, G. (2014). PLUMED 2: New feathers for an old bird. Computer Physics Communications, 185(2), 604–613. https://doi.org/10.1016/j.cpc.2013.09.018
12. Abraham, M. J., Murtola, T., Schulz, R., Páll, S., Smith, J. C., Hess, B., & Lindahl, E. (2015). GROMACS: High performance molecular simulations through multi-level parallelism from laptops to supercomputers. SoftwareX, 1–2, 19–25. https://doi.org/10.1016/j.softx.2015.06.001
