# Supplementary material for: Protonation-dependent substrate release in a bacterial homolog of vesicular glutamate
Source: Biophys J. 2026 Feb 21;125(7):1565–9. doi: 10.1016/j.bpj.2026.02.027 (PMC13351989; doi:10.1016/j.bpj.2026.02.027)
Supplement: Document S2. Article plus supporting material [file mmc2.pdf]

Biophysical Journal
Biophysical letter

Biophysical Society

Protonation-dependent substrate release in a bacterial homolog of vesicular glutamate

Charles Plate,[1] Natalia Dmitrieva,[2] Samira Gholami,[2,3] Mercedes Alfonso-Prieto,[3] Sanket A. Deshmukh,[1] Davide Mandelli,[3,*] Paolo Carloni,[3,4,*] and Christoph Fahlke[2]

[1]Department of Chemical Engineering, Virginia Tech, Blacksburg, Virginia; [2]Institute of Biological Information Processing (IBI-1) Molecular and Cell Physiology, Forschungszentrum Jülich, Jülich, Germany; [3]Institute of Neuroscience and Medicine (INM-9) Computational Biomedicine, Forschungszentrum Jülich, Jülich, Germany; and [4]Department of Physics, RWTH Aachen University, Aachen, Germany

ABSTRACT The SLC17 family contains diverse organic anion transporters with various stoichiometries and ion coupling mechanisms. A bacterial protein of this family, the D-galactonate transporter DgoT, co-transports two protons per substrate molecule. Although the overall transport cycle of DgoT has been proposed, the role of substrate protonation during its release remains unclear. Galactonate is expected to bind in a deprotonated form due to its low pKa; however, it can be released from the transporter in either a protonated or deprotonated state. In this study, we used well-tempered funnel metadynamics simulations to investigate the microscopic mechanisms underlying protonated and deprotonated galactonate dissociation from the inward-facing, gate-open conformation of DgoT. Our free energy profiles reveal that, although substrate protonation lowers the energy barrier for release and may enhance dissociation kinetics, deprotonated galactonate can also dissociate, albeit less frequently. These findings indicate that galactonate protonation facilitates, but is not strictly required for, substrate release by the bacterial organic anion transporter DgoT.

SIGNIFICANCE The sugar-like molecule galactonate can be used by bacteria as a carbon source thanks to the D-galactonate transporter DgoT. Understanding its proton-coupled transport mechanism includes determining the protonation state of galactonate during substrate release to the cytoplasm. Simulations performed here show that galactonate is more easily released when it carries an extra proton, but it can still dissociate from the transporter without it. Thus, protonation of the titratable substrate emerges as an additional tactic for effective coupled transport.

INTRODUCTION

Secondary active transporters utilize ion gradients to drive the movement of solutes across biological membranes, often operating with strict transport stoichiometries (1), which are ensured by the combination of the stoichiometric binding of several substrates and the controlled isomerization between inward- and outward-facing states (2). The SLC17 family of multifunctional secondary anion active transporters belong to the major facilitator superfamily (MFS) (3). It encompasses organic anion transporters that accumulate neurotransmitters in secretory vesicles (the vesicular glutamate (SLC17A6-8/VGLUT1-3) and nucleotide (SLC17A9/VNUT) transporters), remove carboxylated monosaccharides from lysosomes (SLC17A5/sialin), or extrude organic anions from the kidneys and the liver (SLC17A1-4/NPT1-4) (4). Diverse SLC17 transporters differ in transport coupling: VGLUTs function as H^+-glutamate exchangers (5), sialin is an electroneutral H^+-monosaccharide symporter (6), and VNUTs have been reported to function as ATP uniporters. Thus far, structural information has been obtained from VGLUT2 (7), sialin (8), and the bacterial model protein DgoT (9); the latter is the main D-galactonate (GAL) uptake carrier in *E. coli* (10), co-transporting two protons per one GAL. The three proteins feature 12 transmembrane (TM) helices that are organized into two symmetrical domains, each with six helices (see Fig. S1), which facilitate substrate binding and transport through an alternating-access mechanism (9,11).

Submitted September 26, 2025, and accepted for publication February 19, 2026.

*Correspondence: d.mandelli@fz-juelich.de or p.carloni@fz-juelich.de

Samira Gholami's present address is Institute of Nanotechnology, Karlsruhe Institute of Technology, Karlsruhe, Germany

Editor: Lalima Madan.

https://doi.org/10.1016/j.bpj.2026.02.027

© 2026 The Author(s). Published by Elsevier Inc. on behalf of Biophysical Society.
This is an open access article under the CC BY license (http://creativecommons.org/licenses/by/4.0/).

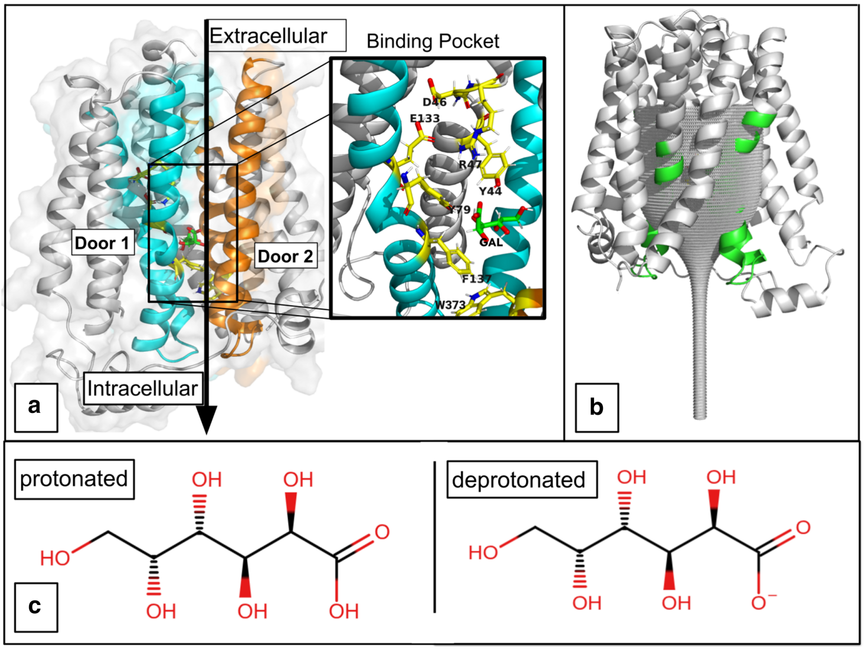

FIGURE 1 DgoT model used in our funnel metadynamics simulations, along with the structures of protonated and deprotonated galactonate. (*a*) Structure of DgoT (ribbon representation), with notable binding canal residues shown in yellow and the substrate (GAL) in green. The gating door 1 is formed by TM2 and TM3 in cyan, and door 2 is formed by TM8 and TM9 in orange, whereas TM11 was removed to ease visualization. (*b*) Funnel restraint volume overlaid on DgoT. Residues 139–158 (belonging to door 1) and residues 373–392 (door 2) are colored in lime and used to define the intracellular gate, which is restrained in an "open" state. (*c*) Structure of galactonate in its protonated and deprotonated states.

We recently described the entire DgoT transport cycle with a combination of classical molecular dynamics (MD) simulations, Markov state modeling, and hybrid quantum mechanical/molecular mechanics MD simulations with experimental approaches (12). DgoT exhibits only two titratable residues in the transmembrane domain, D46 and E133 (Fig. S1); protonation of both acidic residues in the outward-facing conformation opens the extracellular gate and results in binding of galactonate (states 1–3 in Fig. S2). GAL is expected to bind in a deprotonated form because of its low pKa (3.39) in solution. After substrate association, the extracellular gate closes, facilitating transporter transition into an inward-facing occluded conformation (states 4–5 in Fig. S2). In this conformation, deprotonation of D46 (states 5–6 in Fig. S2) results in an open intracellular gate that permits GAL release. As D46 is sequestered from the intracellular solution but can rotate to approach E133, its deprotonation is likely to occur in a stepwise manner through E133 (Fig. S3). Initial proton release from E133, either to the intracellular solvent or to galactonate, is followed by H^+ transfer from D46 to E133. After substrate dissociation, the second co-transported H^+ is released from E133, resulting in intracellular gate closure and return of the empty transporter to an initial outward-facing conformation (states 7–8 in Fig. S2).

Our previous QM/MM simulations showed that the first released H^+ can feasibly protonate GAL. However, spontaneous substrate release from the gate-open conformation in the D46[−]/E133[H] state (state 6 in Fig. S2) was observed for both deprotonated and protonated GAL in unbiased MD simulations (12). Therefore, it remains an open question whether GAL protonation is a crucial step in its dissociation. To obtain further insights into this key aspect, here, we performed enhanced sampling simulations. Specifically, we performed well-tempered funnel metadynamics (13,14) at a physiological temperature of 310.15K to investigate the diffusion of GAL in and out of the binding pocket (Fig. 1 *a*), using the funnel axis as our release coordinate (see supporting material methods for details). Moreover, to avoid closing of the intracellular gate upon substrate dissociation, we restrained it in its open position (Fig. 1 *b*). This choice is justified by extensive validation tests presented in the supporting material, where unbiased MD simulations show that the open intracellular gate is stable on the 500-ns timescale in the D46[-]/E133H state. By comparing the free energy profiles obtained with either deprotonated or protonated GAL (see Fig. 1 *c*), we uncovered kinetic and thermodynamic signatures that distinguish the more favorable substrate protonation state for spontaneous unbinding. Unbiased MD simulations, used to inform the construction of our metadynamics protocol, were consistent with the enhanced sampling results (see supporting material).

RESULTS AND DISCUSSION

Fig. 2 reports the obtained 1D free energy profiles, with convergence tests shown in Figs. S4–S6. Energy barriers for release—defined as the highest energy point along the release pathway (marked 2) compared with the bound state (1)—are 8.2 ± 0.00 kJ/mol for deprotonated GAL and 5.7 ± 0.16 kJ/mol for protonated GAL. Although not staggering, the barrier height difference of 2.5 ± 0.16 kJ/mol between the two states suggests approximately 2.6 times

faster release in the protonated state compared with the deprotonated state, according to Arrhenius kinetics. Evidence from unbiased MD simulations is consistent with the faster release for protonated galactonate, though the deprotonated form can also dissociate. Specifically, protonated galactonate was released in all four of four independent unbiased MD simulations within 500 ns, whereas release of the deprotonated substrate was observed in only two of four simulations of equal length (see Fig. S7). Moreover, the fully converged free energy of release, from the bound state (region around point 1; see also Fig. 3, *a* and *b*) to the unbound state (region around point 3; see also Fig. S10), for the protonated (3.4 $\pm$ 0.00 kJ/mol) and deprotonated state (3.0 $\pm$ 0.00 kJ/mol), turned out to be similar within the uncertainty of the simulations (<1%; see Fig. S5). Thus, the release process appears to be thermodynamically equivalent in the two protonation states. Such small free energy difference is consistent with both protomers being able to dissociate from the transporter, as observed in the unbiased classical MD simulations (Figs. S7–S9), as well as with the previous QM/MM MD simulations showing that the co-transported proton can be released either bound to galactonate or through water molecules (12). In contrast, the lower energy barrier observed for the protonated substrate points to protonation as a kinetic facilitator rather than a thermodynamic requirement. Overall, our results allow us to suggest that substrate protonation acts as a kinetic accelerator—affecting the rate but not the thermodynamic driving force—of substrate release in the inward-facing, open-gate conformation of DgoT. In other words, galactonate protonation provides kinetic control on substrate release without altering its thermodynamic driving force, thus allowing the transporter to ensure substrate dissociation regardless of its protonation state.

The difference in energy barriers can be largely attributed to deprotonated GAL being withheld in the transport canal by a salt bridge with R47 and ion-dipole interactions with the neighboring tyrosines Y44 and Y79 (Figs. 3 *a*, 4 *a*, and S11). Interaction with R47 in the bound state is more frequent (Figs. 4 and S11) and less flexible (Fig. S12, z coordinate below 0.5 nm) for the deprotonated galactonate form, resulting in the anionic substrate being less mobile in the binding site than the neutral form. Upon the substrate leaving the binding site, the behavior is inverted, with deprotonated galactonate showing larger fluctuations in the distance with R47. This allows us to suggest that R47 is the primary determinant of the bound-state stability for the anionic form, as well as the slightly higher barrier for deprotonated GAL. Attempting exit from these interactions, negatively charged GAL was immediately blocked by the bulky aromatic residue W373 that lines the intracellular gate (Fig. 3 *c*). Instead, the protonated (neutral) form of galactonate ceased to form comparable interactions with these crucial binding pocket residues (Figs. 3 *b*, 4 *b*, and S11). Although the contact between the hydrophobic W373 and

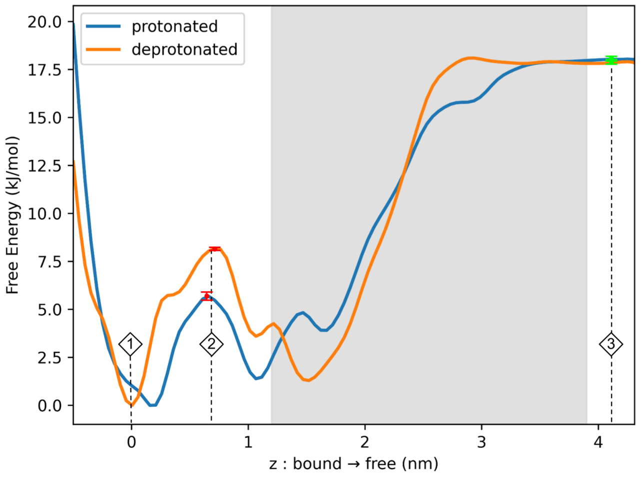

FIGURE 2 Free energy associated with the release of protonated (*blue*) and deprotonated (*orange*) GAL plotted as a function of its distance *z* from the binding pocket along the funnel axis. The shaded region represents the uncertain portion of the free energy: here, GAL interacts with both the funnel wall and the protein (see supporting material methods for details). Numbered diamonds denote characteristic points along the GAL release pathway: (1) bound (see structure in Fig. 3, *a* and *b*); (2) gate crossing barrier (see structure in Fig. 3, *c* and *d*); (3) fully solvated, unbound (see structure in Fig. S10). Error bars represent the standard deviations over the final 1000 ns of either metadynamics simulation for the (*red*) energy barrier from (1) to (2) and (*lime*) free energy of release from (1) to (3). The free energy of release extracted from the free energy profile must be corrected by applying the entropic funnel correction of −14.4 kJ/mol, as determined for our specific simulation setup (see Fig. S5).

the highly polar galactonate is unfavorable, it is less disadvantageous for the substrate neutral form (Fig. S13), resulting in overall smoother release for protonated galactonate. Our findings are consistent with the essential role of R47 in binding of the GAL carboxylic group evidenced by SSME experiments on the R47Q mutant (12).

The preferential release of protonated D-galactonate shown here and the regulation of intracellular gate opening by substrate protonation suggested by previous unbiased MD simulations (12) provide a possible explanation for a surprising set of recent mutagenesis results (12). In D46N DgoT, H^+ transfer from E133 to protonate galactonate is still possible, but the resulting D46N/E133[-] state will exhibit a closed intracellular gate, in line with the observed lack of GAL transport. Instead, in E133Q DgoT, GAL protonation is precluded, but the intracellular gate can oscillate between closed and open states, clarifying why this mutant does not show net transport but still permits GAL exchange. Lastly, in D46N/E133Q DgoT, galactonate remains deprotonated, but a less tightly regulated intracellular gate opening will permit galactonate dissociation in either direction, explaining the observed GAL homoexchange.

During evolution, multiple mechanisms have developed to ensure strict stoichiometric transport in secondary active

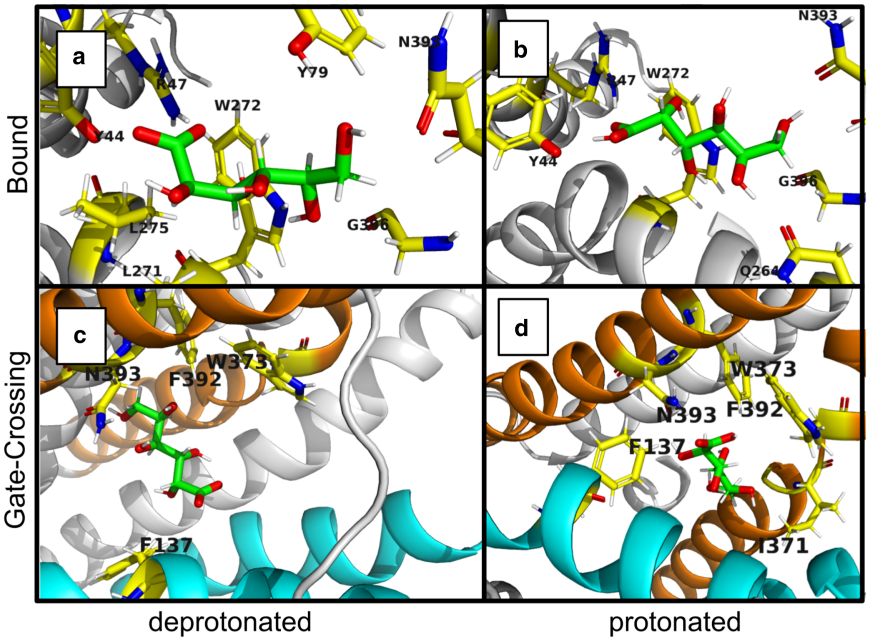

FIGURE 3 Representative structures of DgoT extracted from state 1 (bound) and state 2 (gate-crossing), as defined in Fig. 2. (a) Bound deprotonated galactonate. (b) Bound protonated galactonate. (c) Gate-crossing deprotonated galactonate. (d) Gate-crossing protonated galactonate. The protein frame is in gray, except door 1 and door 2 of the gate, which are colored cyan and orange, respectively. Residues exhibiting 3.5-Å contacts with galactonate are highlighted in yellow, and GAL is colored in lime. Snapshots for state 3 (unbound) in Fig. 2 are shown in the supporting material (see Fig. S10).

transporters. There exist stoichiometrically coupled binding, translocation between outward- to inward-facing conformations, or exclusive release at certain binding stoichiometry. In H^+-coupled transporters, protonation of the titratable substrate as part of the transport cycle emerges as an additional tool for effective and coupled transport (15). Here we suggest that, although galactonate protonation is dispensable, it may enhance the kinetics of substrate release in the inward-facing, open-gate conformation of D46[-]/E133[H] DgoT.

Besides DgoT, SLC17 family encompasses other proton-coupled organic anion transporters (4), including the H^+-glutamate exchangers VGLUTs (5) and the H^+-sialic acid symporter sialin (8). The different proton coupling mechanisms across SLC17 members have been attributed to their different number and arrangement of protein titratable residues (Fig. S1). The results presented here and in (12) suggest that the organic anion substrate may also contribute to proton coupling, provided that it is titratable. VGLUTs transport glutamate into the acidic lumen of the synaptic vesicle, and the pKa values in bulk solution of galactonate (3.39) and glutamate (4.07–4.25) are within 1 pH unit, suggesting that the substrate could potentially be released in its protonated form also in VGLUTs. In contrast, substrate

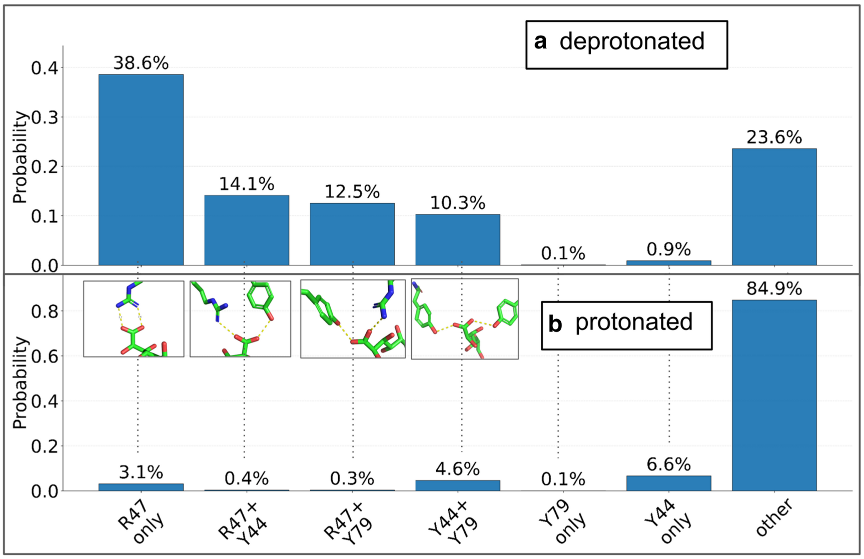

FIGURE 4 Percentage of bound frames exhibiting GAL-carboxyl interactions with specific residues, binned into discrete states. R47-only interaction comprises the first and second peaks of the R47-GAL (C-guanidinium to C-carboxyl) distance probability distribution representing the bidentate and monodentate contact modes, which extend to 5.0 Å (Fig. S14). Y44 and Y79 hydrogen bonds were classified based on the first peak of the GAL-Tyr (C-carboxyl to O-phenol) distance probability distribution up to 4.2 Å (Fig. S15). Panels (*a*) and (*b*) show the results of this analysis for deprotonated and protonated galactonate, respectively.

protonation is less likely in sialin, as sialic acid is more acidic (pKa = 2.60) than either glutamate or galactonate. Computational and experimental studies are needed to assess whether substrate protonation is a conserved strategy to facilitate substrate release across proton-coupled SLC17 transporters.

ACKNOWLEDGMENTS

C.P. gratefully acknowledges funding from the "Algorithms & Software for SUpercomputers with emerging aRchitEctures (ASSURE)" NSF-funded International Research Experiences for Students (IRES). D.M. and P.C. acknowledge support by the European Union's HORIZON MSCA Doctoral Networks program, under Grant Agreement No. 101072344, project AQTIVATE (Advanced computing, QuanTum algorIthms and datadriVen Approaches for Science, Technology, and Engineering). This work was also supported by the Deutsche Forschungsgemeinschaft (German Research Foundation) to C.F. (FA 301/15–2), P.C. (CA 973/27-2), and M.A.-P. (AL 2511/1-2) as part of Research Unit FOR 2518, DynIon. The authors gratefully acknowledge the Advanced Research Computing (ARC) facility at Virginia Tech, and computing time on the supercomputer JURECA (16) at Forschungszentrum Jülich under grant no. dgot-gal.

DECLARATION OF INTERESTS

The authors declare no competing interests.

SUPPORTING CITATIONS

References (17–26) appear in the supporting material.

SUPPORTING MATERIAL

Supporting material can be found online at https://doi.org/10.1016/j.bpj.2026.02.027.

REFERENCES

1. Drew, D., and O. Boudker. 2024. Ion and lipid orchestration of secondary active transport. *Nature*. 626:963–974. https://doi.org/10.1038/s41586-024-07062-3.
2. Forrest, L. R., R. Krämer, and C. Ziegler. 2011. The structural basis of secondary active transport mechanisms. *Biochim. Biophys. Acta Bioenerg.* 1807:167–188. https://doi.org/10.1016/j.bbabio.2010.10.014.
3. Drew, D., R. A. North, …, M. Tanabe. 2021. Structures and General Transport Mechanisms by the Major Facilitator Superfamily (MFS). *Chem. Rev.* 121:5289–5335. https://doi.org/10.1021/acs.chemrev.0c00983.
4. Reimer, R. J. 2013. SLC17: A functionally diverse family of organic anion transporters. *Mol. Aspect. Med.* 34:350–359. https://doi.org/10.1016/j.mam.2012.05.004.
5. Kolen, B., B. Borghans, …, C. Fahlke. 2023. Vesicular glutamate transporters are H^+-anion exchangers that operate at variable stoichiometry. *Nat. Commun.* 14:2723. https://doi.org/10.1038/s41467-023-38340-9.
6. Morin, P., C. Sagné, and B. Gasnier. 2004. Functional characterization of wild-type and mutant human sialin. *EMBO J.* 23:4560–4570. https://doi.org/10.1038/sj.emboj.7600464.
7. Li, F., J. Eriksen, …, R. M. Stroud. 2020. Ion transport and regulation in a synaptic vesicle glutamate transporter. *Science*. 368:893–897. https://doi.org/10.1126/science.aba9202.
8. Hu, W., C. Chi, …, H. Zheng. 2023. The molecular mechanism of sialic acid transport mediated by Sialin. *Sci. Adv.* 9:eade8346. https://doi.org/10.1126/sciadv.ade8346.
9. Leano, J. B., S. Batarni, …, R. H. Edwards. 2019. Structures suggest a mechanism for energy coupling by a family of organic anion transporters. *PLoS Biol.* 17:e3000260. https://doi.org/10.1371/journal.pbio.3000260.
10. Deacon, J., and R. A. Cooper. 1977. D-galactonate utilisation by enteric bacteria The catabolic pathway in Escherichia coli. *FEBS Lett.* 77:201–205. https://doi.org/10.1016/0014-5793(77)80234-2.
11. Li, F., J. Eriksen, …, R. H. Edwards. 2022. Diversity of function and mechanism in a family of organic anion transporters. *Curr. Opin. Struct. Biol.* 75:102399. https://doi.org/10.1016/j.sbi.2022.102399.
12. Dmitrieva, N., S. Gholami, …, C. Fahlke. 2024. Transport mechanism of DgoT, a bacterial homolog of SLC17 organic anion transporters. *EMBO J.* 43:6740–6765. https://doi.org/10.1038/s44318-024-00279-y.
13. Limongelli, V., M. Bonomi, and M. Parrinello. 2013. Funnel metadynamics as accurate binding free-energy method. *Proc. Natl. Acad. Sci. USA*. 110:6358–6363. https://doi.org/10.1073/pnas.1303186110.
14. Raniolo, S., and V. Limongelli. 2020. Ligand binding free-energy calculations with funnel metadynamics. *Nat. Protoc.* 15:2837–2866. https://doi.org/10.1038/s41596-020-0342-4.
15. Liu, Y., C. Li, …, G. A. Voth. 2021. Key computational findings reveal proton transfer as driving the functional cycle in the phosphate transporter PiPT. *Proc. Natl. Acad. Sci. USA*. 118:e2101932118. https://doi.org/10.1073/pnas.2101932118.
16. Jülich Supercomputing Centre 2021. JURECA: Data Centric and Booster Modules implementing the Modular Supercomputing Architecture at Jülich Supercomputing Centre. *Journal of large-scale research facilities*. 7:A182. https://doi.org/10.17815/jlsrf-7-182.
17. Klauda, J. B., R. M. Venable, …, R. W. Pastor. 2010. Update of the CHARMM All-Atom Additive Force Field for Lipids: Validation on Six Lipid Types. *J. Phys. Chem. B*. 114:7830–7843. https://doi.org/10.1021/jp101759q.
18. Huang, J., S. Rauscher, …, A. D. MacKerell. 2017. CHARMM36m: an improved force field for folded and intrinsically disordered proteins. *Nat. Methods*. 14:71–73. https://doi.org/10.1038/nmeth.4067.
19. Jorgensen, W. L., J. Chandrasekhar, …, M. L. Klein. 1983. Comparison of simple potential functions for simulating liquid water. *J. Chem. Phys.* 79:926–935. https://doi.org/10.1063/1.445869.
20. Zoete, V., M. A. Cuendet, …, O. Michielin. 2011. SwissParam: A fast force field generation tool for small organic molecules. *J. Comput. Chem.* 32:2359–2368. https://doi.org/10.1002/jcc.21816.
21. Essmann, U., L. Perera, …, L. G. Pedersen. 1995. A smooth particle mesh Ewald method. *J. Chem. Phys.* 103:8577–8593. https://doi.org/10.1063/1.470117.
22. Bussi, G., D. Donadio, and M. Parrinello. 2007. Canonical sampling through velocity rescaling. *J. Chem. Phys.* 126:014101. https://doi.org/10.1063/1.2408420.
23. Parrinello, M., and A. Rahman. 1981. Polymorphic transitions in single crystals: A new molecular dynamics method. *J. Appl. Phys.* 52:7182–7190. https://doi.org/10.1063/1.328693.
24. Barducci, A., G. Bussi, and M. Parrinello. 2008. Well-Tempered Metadynamics: A Smoothly Converging and Tunable Free-Energy Method. *Phys. Rev. Lett.* 100:020603. https://doi.org/10.1103/physrevlett.100.020603.
25. Tribello, G. A., M. Bonomi, …, G. Bussi. 2014. PLUMED 2: New feathers for an old bird. *Comput. Phys. Commun.* 185:604–613. https://doi.org/10.1016/j.cpc.2013.09.018.
26. Abraham, M. J., T. Murtola, …, E. Lindahl. 2015. GROMACS: High performance molecular simulations through multi-level parallelism from laptops to supercomputers. *SoftwareX*. 1–2:19–25. https://doi.org/10.1016/j.softx.2015.06.001.

Biophysical Journal, Volume *125*

Supplemental information

Protonation-dependent substrate release in a bacterial homolog of vesicular glutamate

Charles Plate, Natalia Dmitrieva, Samira Gholami, Mercedes Alfonso-Prieto, Sanket A. Deshmukh, Davide Mandelli, Paolo Carloni, and Christoph Fahlke

Supporting Material for "Protonation-dependent substrate release in a bacterial homolog of vesicular glutamate transporters"

Charles Plate[1], Natalia Dmitrieva[2], Samira Gholami[2,3,†], Mercedes Alfonso-Prieto[3], Sanket A. Deshmukh[1], Davide Mandelli*[3], Paolo Carloni*[3,4], Christoph Fahlke[2]

[1]Department of Chemical Engineering, Virginia Tech, Blacksburg, VA 24061, USA
[2]Institute of Biological Information Processing (IBI-1) Molecular and Cell Physiology, Forschungszentrum Jülich, Wilhelm-Johnen-Straße, 52428 Jülich, Germany
[3]Institute of Neuroscience and Medicine (INM-9) Computational Biomedicine, Forschungszentrum Jülich, Wilhelm-Johnen-Straße, 52428 Jülich, Germany
[4]Department of Physics, RWTH Aachen University, 52056 Aachen, Germany

†Present address: Institute of Nanotechnology, Karlsruhe Institute of Technology, Kaiserstr. 12, 76131 Karlsruhe, Germany

*corresponding author

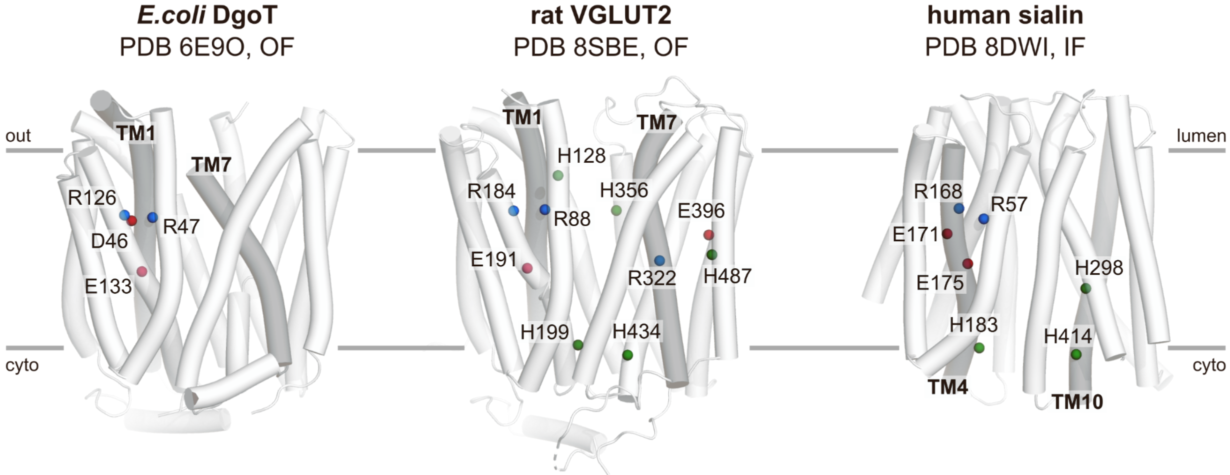

Figure S1. Comparison of DgoT with SLC17 transporters VGLUT2 and sialin. Representative experimental structures are shown with their Protein Data Bank (PDB) code and conformation (inward- or outward-facing, IF and OF, respectively). Gating helices are shown in darker gray, positions of titratable and charged amino acids in the transmembrane region of the respective proteins are shown in red (Asp/Glu), green (His) or blue (Arg/Lys). The horizontal line indicates the approximate location of the membrane and the SLC17 proteins are oriented so that the outside medium ('out) or the vesicular lumen ('lumen') is on top, whereas the cytoplasm is at the bottom.

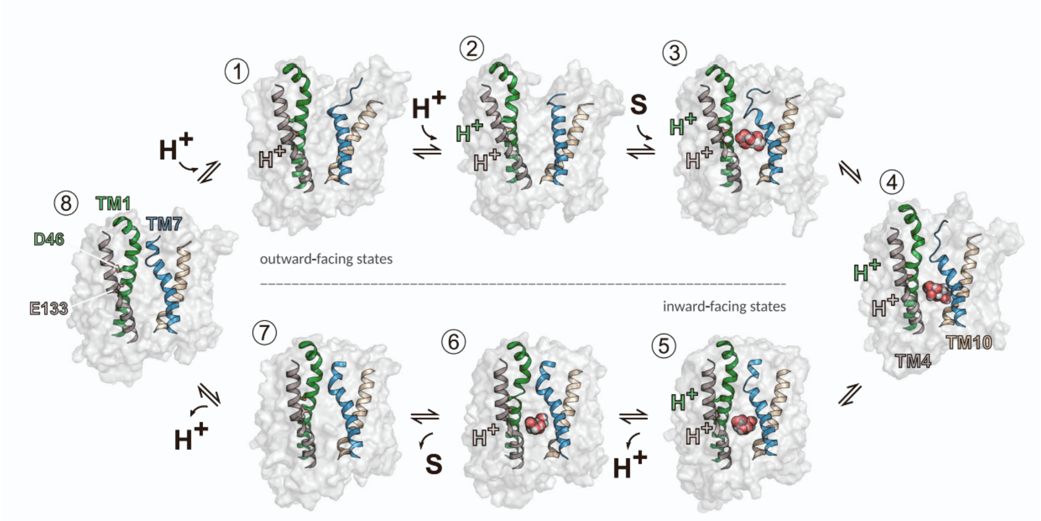

Figure S2: Transport cycle of DgoT (adapted from (1) under a Creative Commons CC-BY 4.0 license). The transport cycle of the bacterial transporter DgoT begins with the protein in an outward-facing conformation (1), where protonation of two acidic residues, D46 and E133, stabilizes the extracellular gate in an open state (2). This configuration allows the anionic substrate D-galactonate to bind from the extracellular side (3). Substrate binding induces conformational changes, leading to closure of the extracellular gate and transition into an occluded state where the substrate binding site becomes inaccessible from both sides of the membrane (4). Subsequently, DgoT undergoes a major conformational shift to an inward-facing state (5), enabling access to the cytoplasmic side. In this state, deprotonation of D46 opens the intracellular gate, permitting the release of galactonate, either in a protonated or deprotonated form (6). After substrate release, the intracellular gate closes, and deprotonation of E133 resets the transporter to its outward-facing conformation (7), ready for another transport cycle (8). In the present study we focus on substrate release from the inward-facing, open-gate conformation shown in (6).

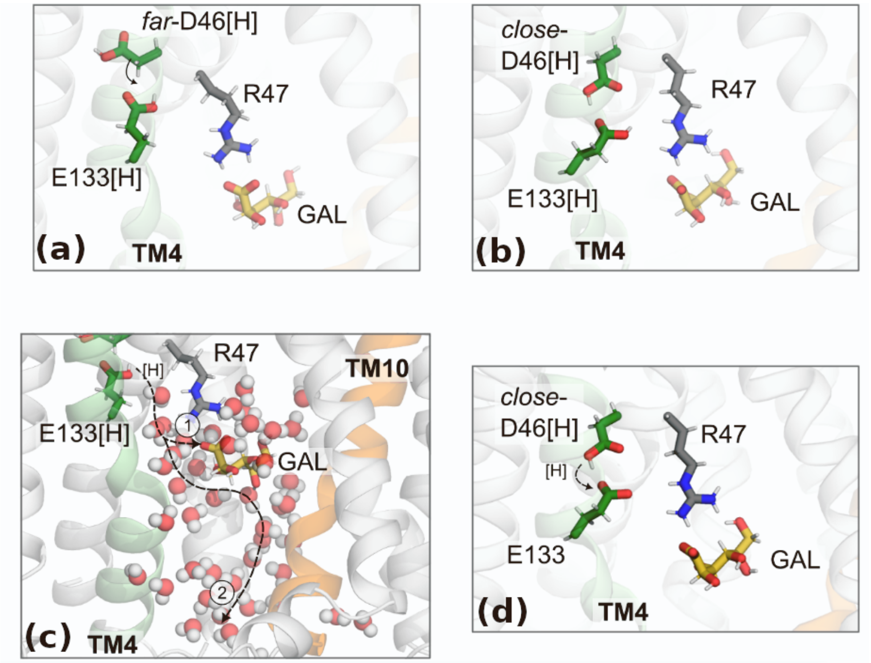

Figure S3: Schematic Illustration of the proton release in DgoT. (a) Initially, both D46 and E133 are protonated in IF DgoT, and side chain of D46 is in the *far* conformation. (b) After a conformational transition of D46[H] from a distant to a close conformation with respect E133 (*far*→*close*) a proton transfer pathway between D46 and E133 is formed. (c) Initial proton transfer from E133 to the nearby water molecule results in subsequent proton transfer either towards the substrate galactonate (pathway 1) or formation of a hydronium ion stabilized within the water network (pathway 2). Gating helices TM4 and TM10 are colored in green and orange, respectively, whereas TM2 and TM11 are hidden to reveal the permeation pathway. (d) Deprotonation of D46 facilitated by the now deprotonated E133, with the galatonate substrate still interacting with R47 before its release.

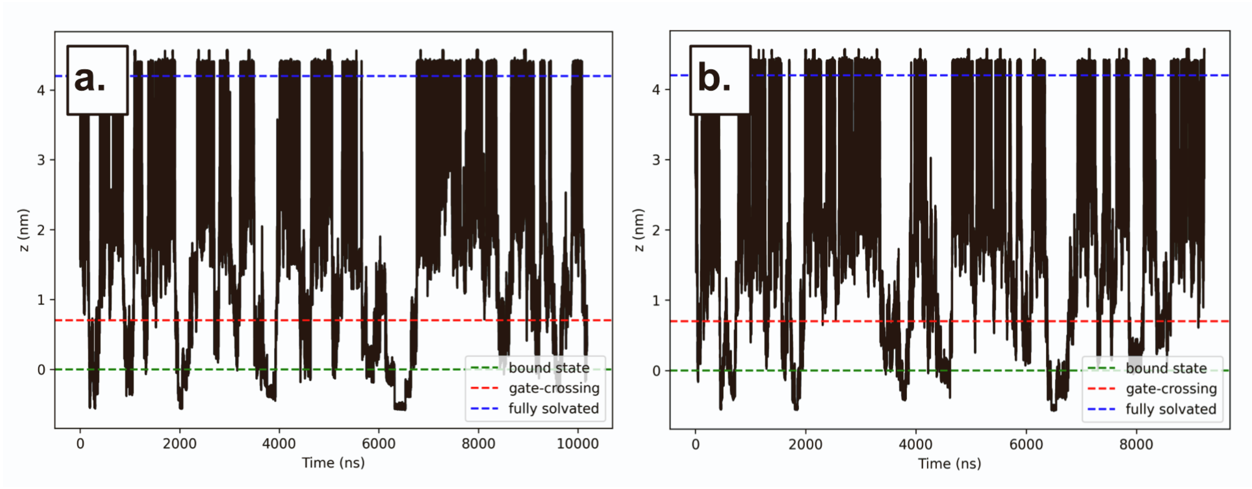

Figure S4: Time evolution of the *z* collective variable in the metadynamics simulation of the *deprotonated* (a) and *protonated* (b) system, showing many transitions between the bound and the unbound state of the substrate during ~10 μs.

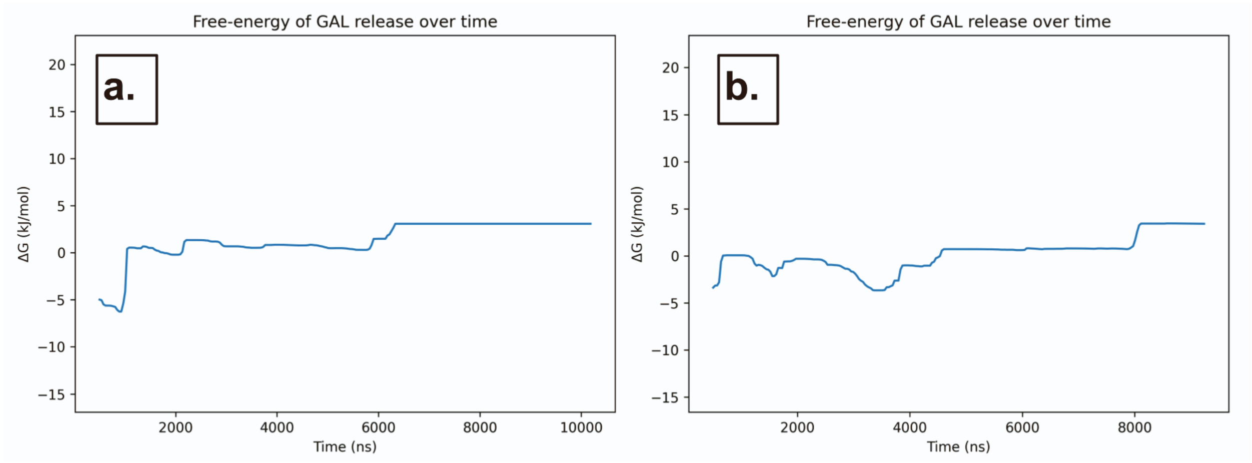

Figure S5: The figure reports the free energy of release, $\Delta G = -k_B T \log\left(\frac{\int_{\Delta_\mathrm{u}} e^{-\beta W(z)} \mathrm{d}z}{\int_{\Delta_\mathrm{b}} e^{-\beta W(z)} \mathrm{d}z}\right) + k_B T \log\left(C_0 \pi R^2 \Delta_u\right)$, between unbound (u) and bound (b) states as a function of time along the metadynamics trajectory, calculated using the instantaneous estimate of the 1D free energy surface W(z). The bound and unbound regions were defined as spanning the z-intervals Δ_b = (-0.1,0.1) nm, Δ_u = (4,4.2) nm for the deprotonated GAL and Δ_b = (0.1,0.3) nm, Δ_u = (4,4.2) nm for protonated GAL. The value of ΔG reported here already includes the entropic correction of $k_B T \log\left(C_0 \pi R^2 \Delta_u\right) =$ -14.4 kJ/mol, where R=0.1 nm is the radius of the cylindrical restraint in the unbound region, and C_0 is the standard concentration. The standard deviation calculated over the final 1000 ns of the trajectory corresponds to a relative error below 1% for both systems.

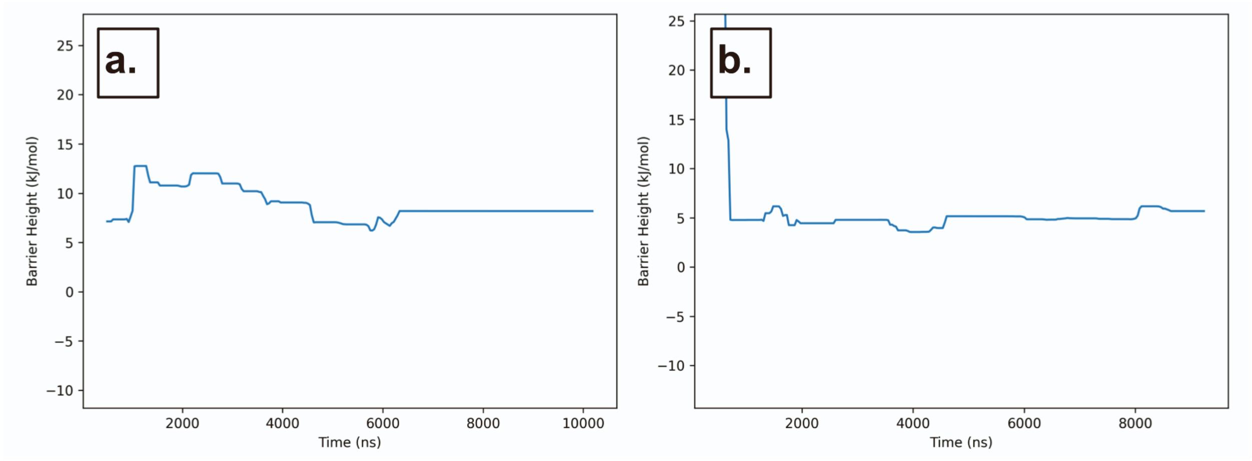

Figure S6: The energy barrier between bound and unbound states for deprotonated GAL (a) and protonated GAL (b), defined here as the free energy difference between the highest and lowest energy point on the directed path from z=-0.25 to z=1.2 nm. The standard deviation calculated over the final 1000 ns of the trajectory corresponds to a relative error of <1% for the deprotonated system and 2.7% for the protonated system.

			Replica / GAL Residence Time (ns)				
System	Protonation of GAL	Initial Gate Conformation	1	2	3	4	GAL Release
d-closed	[-]	closed	-1	-1	-1	-1	0/4
d-open	[-]	open	265	-1	39	-1	2/4
p-closed	[H]	closed	-1	-1	-1	-1	0/4
p-open	[H]	open	124	75	37	90	4/4

Figure S7. Residence time for galactonate release. A value of -1 indicates that no substrate release was observed within the duration of the simulation. We compared the release behavior of protonated and deprotonated galactonate in MD simulations of DgoT in its D46[-]/E133[H] protonation state. We considered four system configurations, controlling for the initial conformation of the intracellular gate: protonated (p) GAL with the intracellular gate either open or closed (*p-ope*n and *p-closed*, respectively*)* and deprotonated (d) GAL with the intracellular gate either open or closed (*d-ope*n and *d-closed*). For each system, we performed four independent 500 ns-long replica simulations, starting with the substrate in the binding site and recording its residence time, measured as the time before the ligand's center of mass exceeds z=3.9 nm. We found that while protonated GAL release occurred in all 4 gate-open replicas within the 500 ns trajectory length, deprotonated GAL dissociation was observed in only 2 out of 4. Notably, in simulations initiated with a closed gate (p-closed and d-closed), the intracellular gate remained stably closed in all four replicas. Likewise, simulations initiated with an open gate (p-open and d-open) remained predominantly open in all four replicas. This behavior suggests bimodality of gate conformation for the D46[-]/E133[H] protonation state, with an energy barrier larger than k_bT separating the open and closed states. Although the current sampling does not allow for a quantitative estimate of the substrate residence time, the results indicate that the protonation state of galactonate, while potentially facilitating release, is not a strict requirement for dissociation and release of both protonated and deprotonated GAL is feasible.

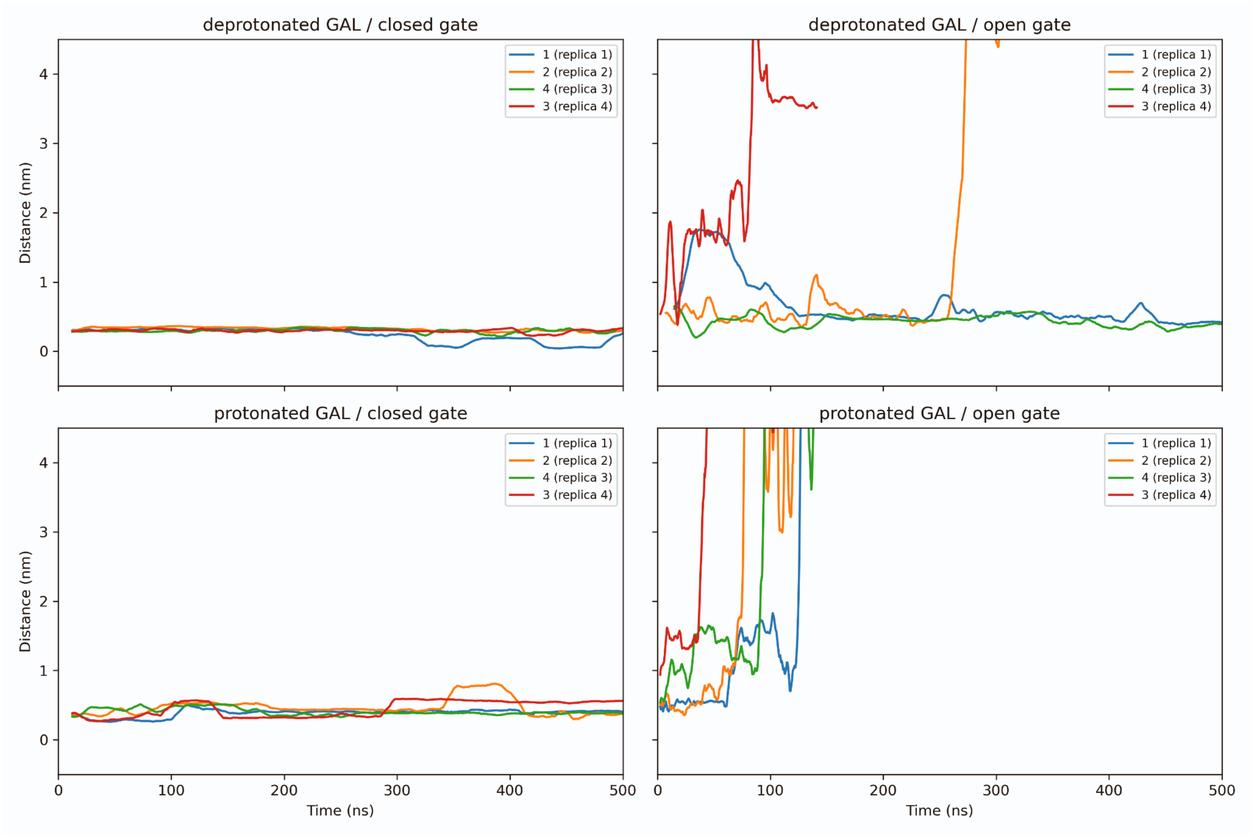

Figure S8: Distance from center of mass of protein to center of mass of galactonate in unbiased molecular dynamics simulations for the four systems investigated, as indicated in the titles of each panel.

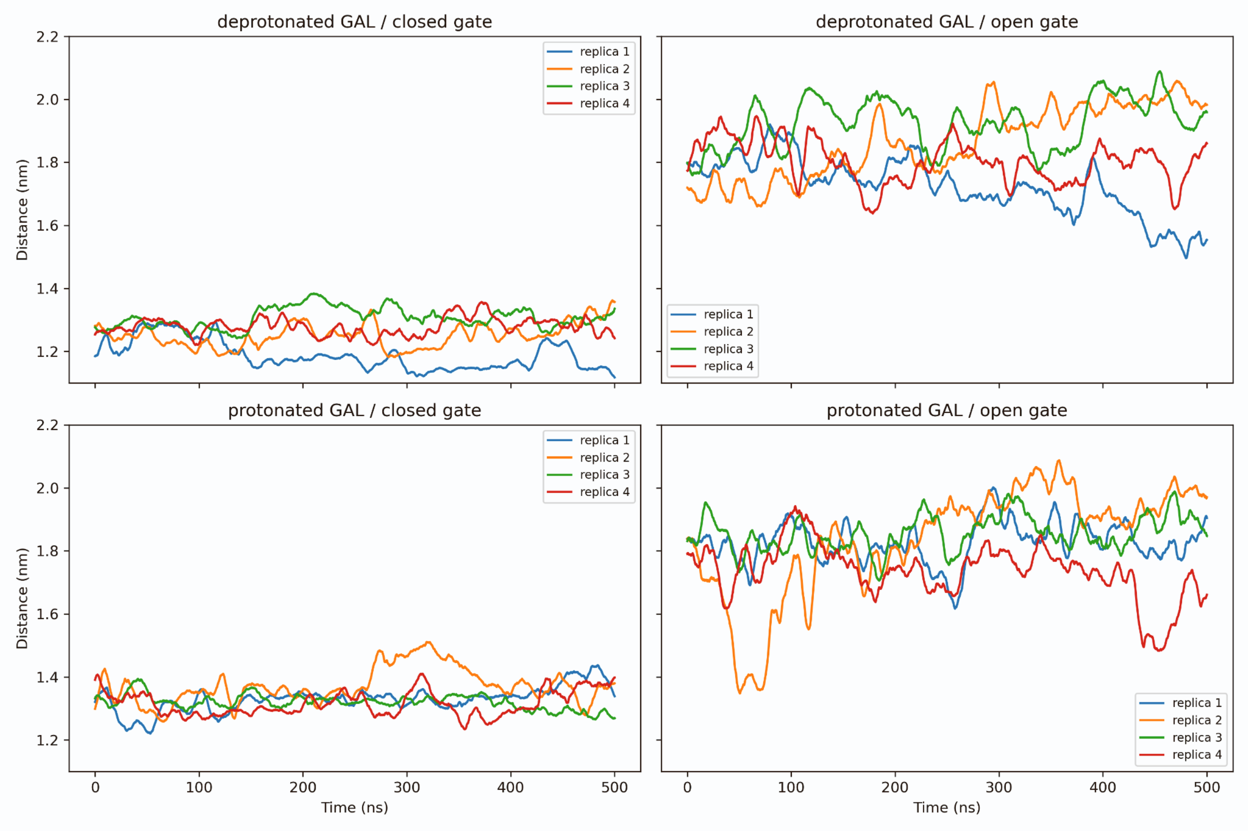

Figure S9: Gate distance during unbiased MD simulations, measured as a center of mass distance between the two "doors" described in the main text (see Fig. 1). Each panel refers to one of the four systems investigated, as indicated in the panel's title.

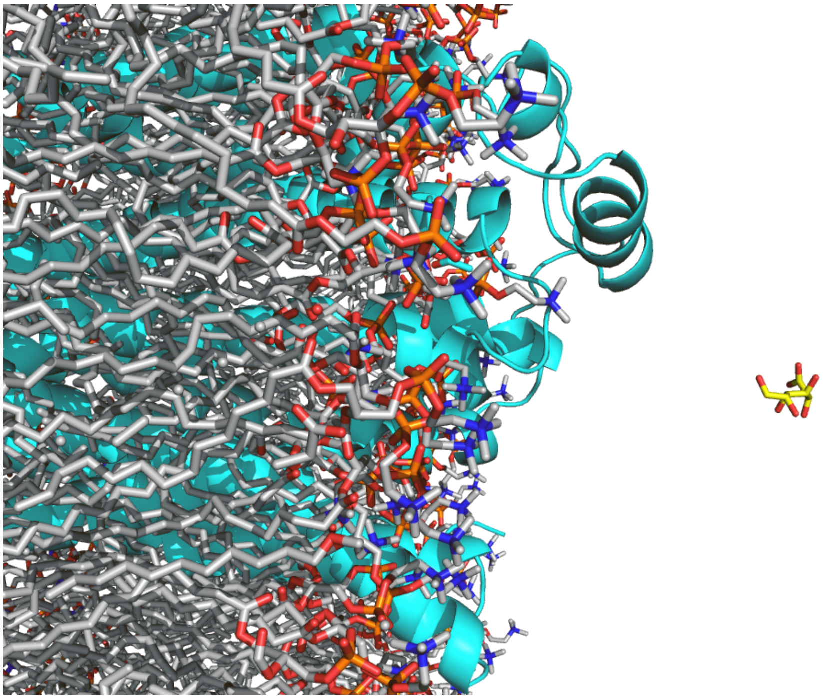

Figure S10: Galactonate Unbound state (point (3) at z=4.25 nm in Fig. 2 in the main text). Galactonate (yellow-carbon), DgoT (cyan cartoon), Lipid Bilayer (gray-carbon).

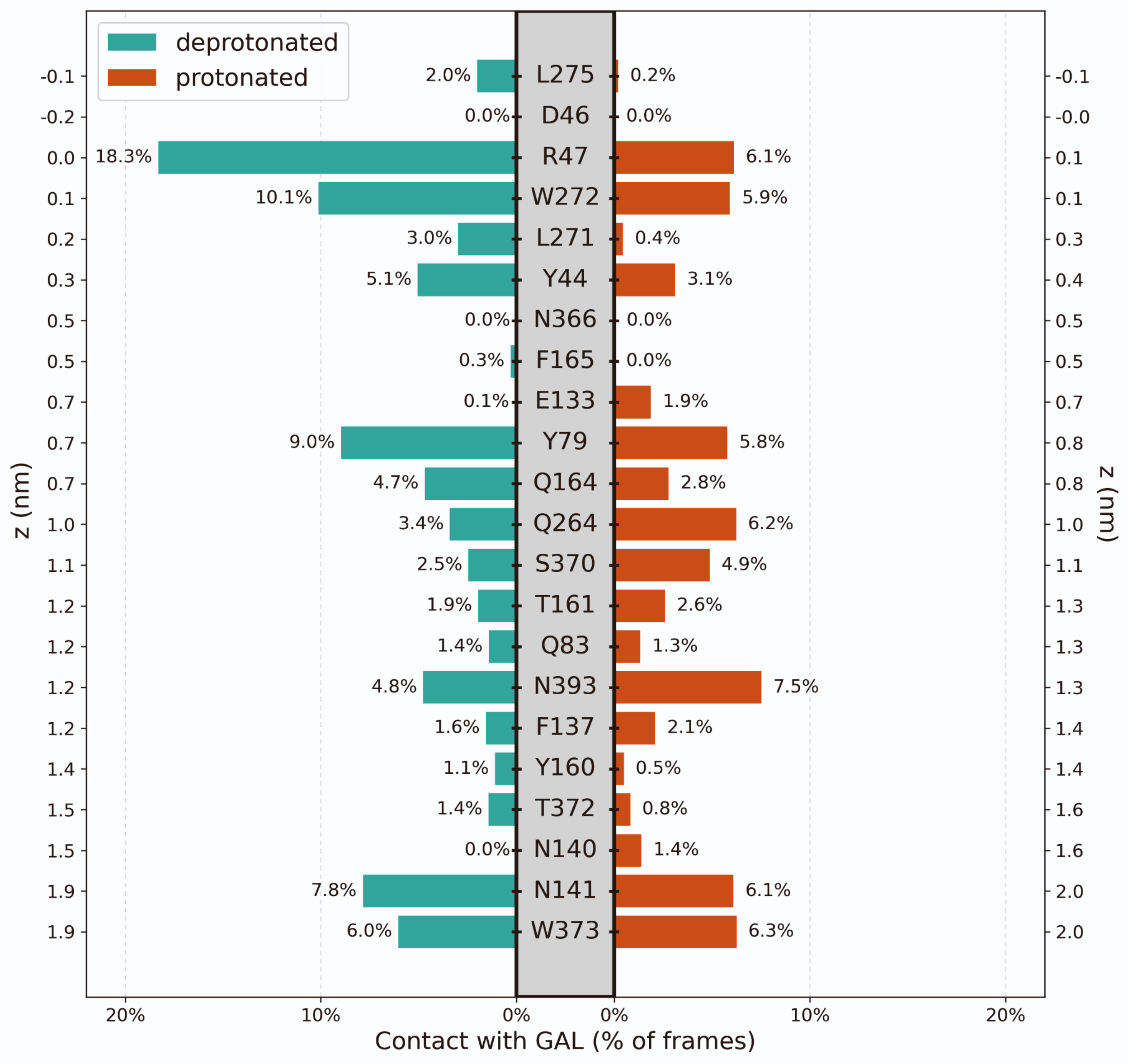

Figure S11: Binding pocket residue contacts with galactonate, using a 3.2 Å all-atom minimum distance. Left and right y-axes portray the average z-coordinate of the center-of-mass of residues oriented on the chart from binding pocket (top) to gate (bottom) during the simulations of deprotonated and protonated GAL.

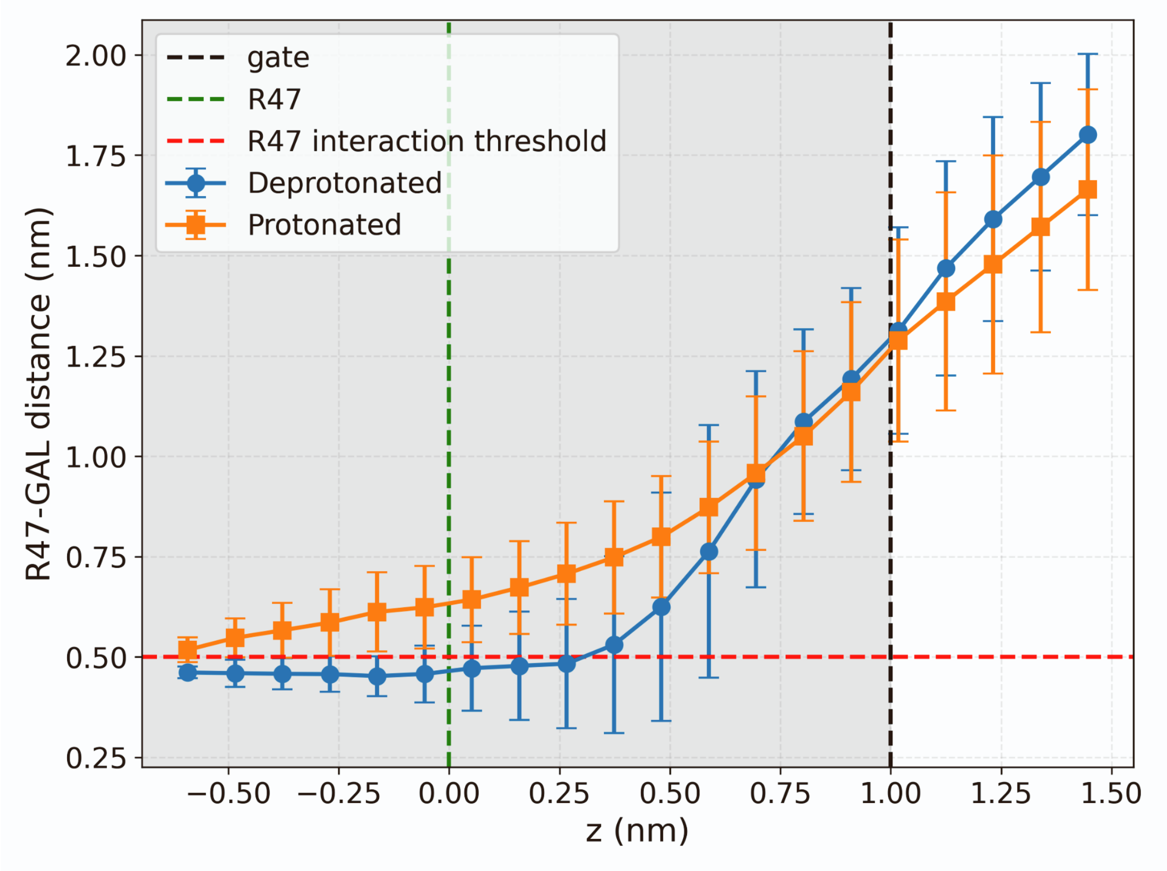

Figure S12. Average R47 C-guanidinium to GAL C-carboxyl with standard deviation, as a function of the reaction coordinate z. Distance values were binned with 0.1 nm binwidth and each mark represents the center of a bin. The black dashed line represents the location of the intracellular gate, whereas the green dashed line shows the location of the R47 residue. The red dashed line indicates an approximate threshold for interaction with R47 (0.50 nm), above which the interaction can be considered weak or broken. The shaded portion of the graph represents the bound state region (i.e. before the intracellular gate).

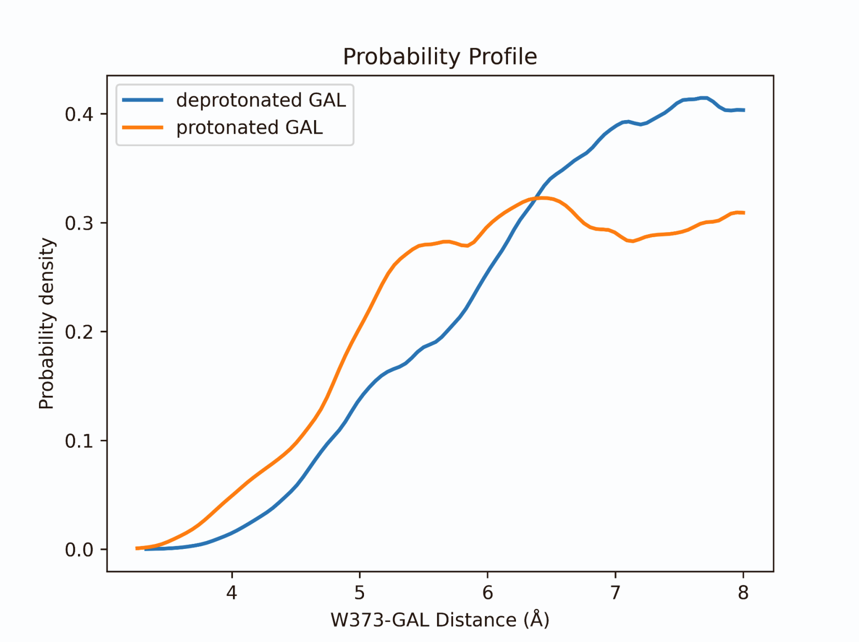

Figure S13: Distance between the center of mass of W373 and the carboxyl carbon of galactonate.

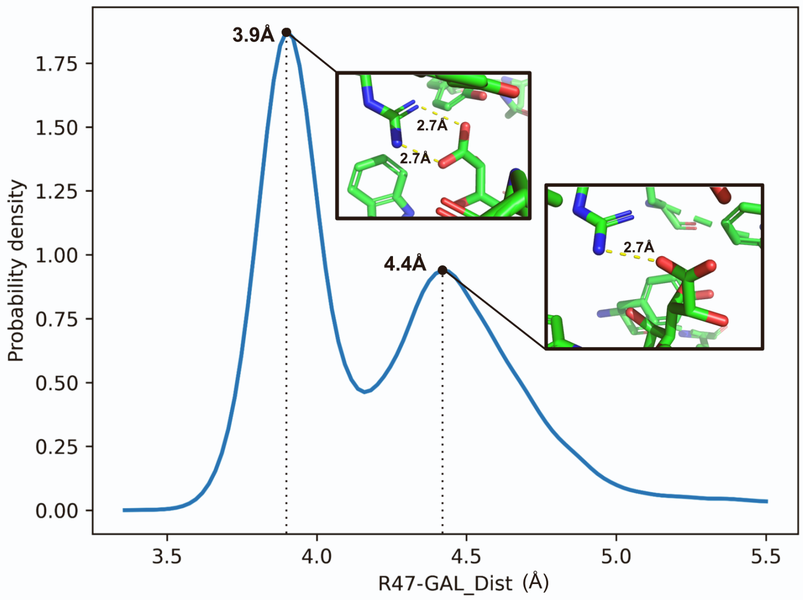

Figure S14: Distance probability distribution measuring the distance *R47 (C-guanidinium*) to *GAL (C-carboxyl)* for the *deprotonated* galactonate simulation. First and second peaks at 3.9 and 4.4 Å show bidentate and monodentate contacts, respectively.

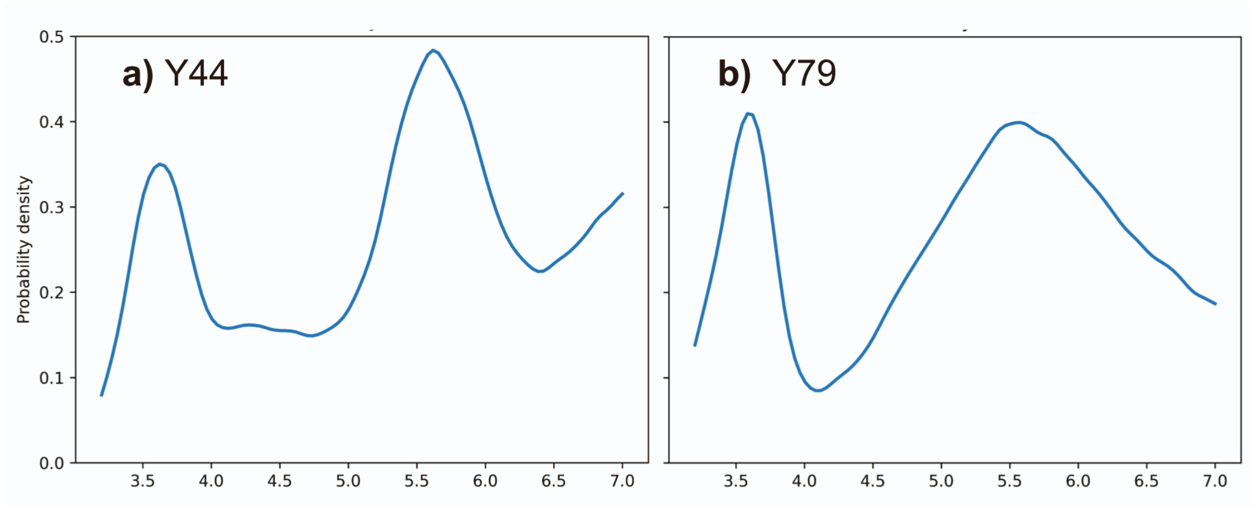

Figure S15: Distance probability distribution between GAL *(C-carboxyl)* and a) Y44 or b) Y79 (C-carboxyl – O-phenol) for the *deprotonated* galactonate simulation. The first peak until 4.2 Å represents a hydrogen bond between GAL and tyrosine.

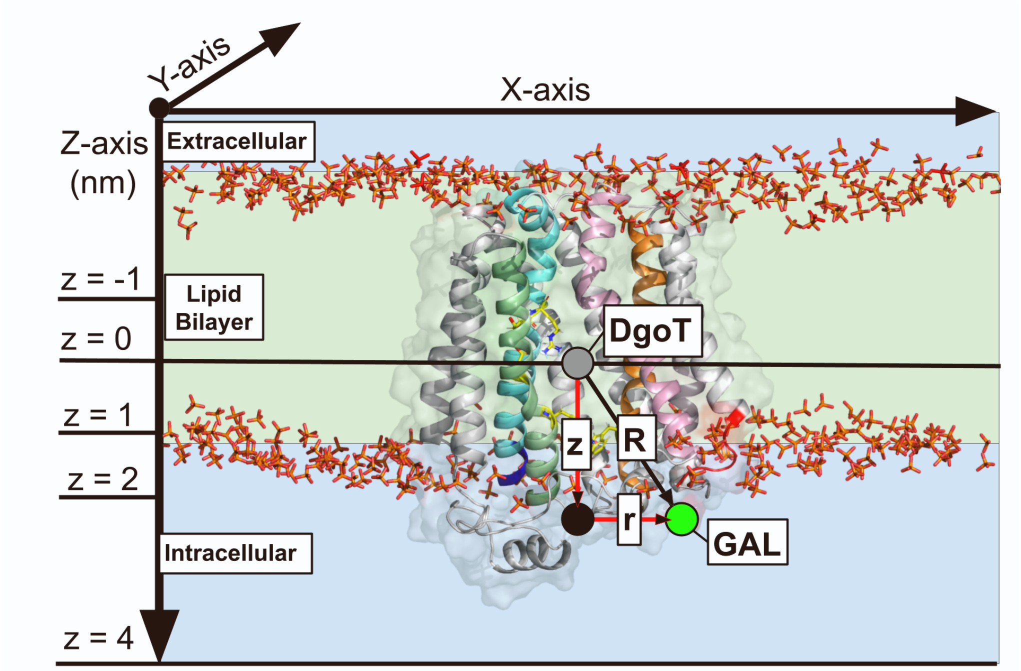

Figure S16: System coordinates: the displacement vector ***R*** is the center of mass of GAL minus the center of mass of DgoT*;* ***z*** is simply the z-component of *R* (R.z) and corresponds roughly to the release coordinate of GAL; ***r*** is the radius used in the funnel restraint calculated as $\sqrt{R.x^2 + R.y^2}$

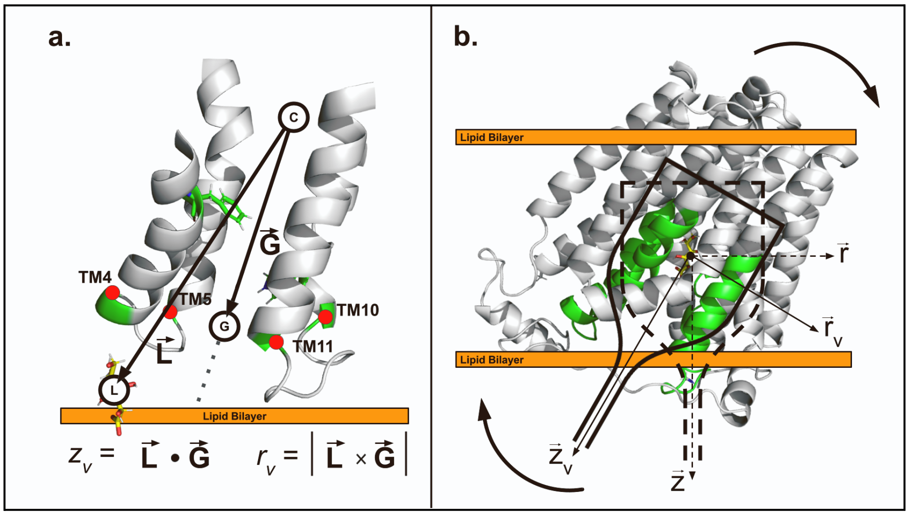

Figure S17: Rotationally invariant collective variables. The vector $\vec{G}$ in panel A is a unit vector directed from the center of mass of the protein *C* to point *G* which denotes the center of the 4 Cα atoms located at the ends of the gating helices TM4 and TM10 (containing the intracellular gate residues F137 and W373 shown as lime sticks), as well as the adjacent helices TM5 and TM11, denoted with red points in the right panel. $\vec{L}$ points from *C* to the center of mass of the ligand *L*. Our final invariant GAL release coordinate z_v (see panel B) is thus given by $\vec{L} \bullet \vec{G}$ and the invariant radius r_v which is necessary to construct the walls of the funnel, is computed as $|\vec{L}$ x $\vec{G}|$.

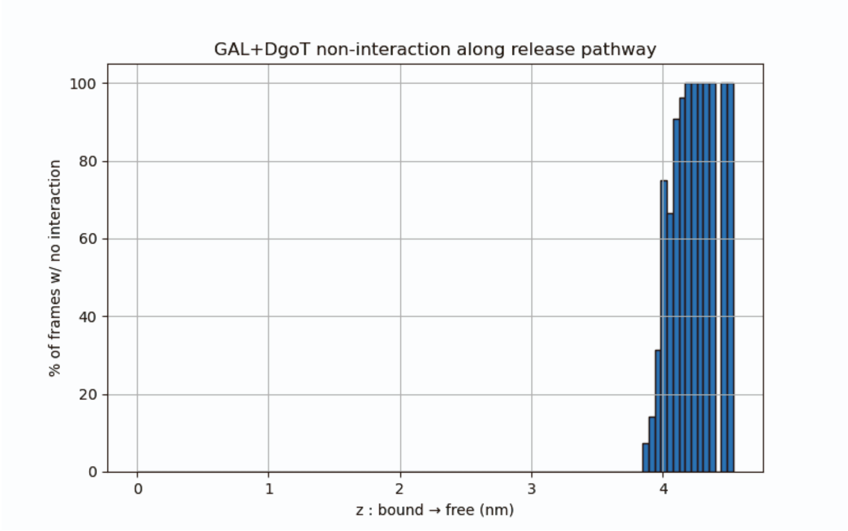

Figure S18: Interaction between DgoT and GAL substrate along the release reaction coordinate. Bars show the percentage of frames with minimum distance between atoms of DgoT and atoms of GAL exceeding 12 Å – the Lennard Jones and Coulomb interaction cutoff of the simulation.

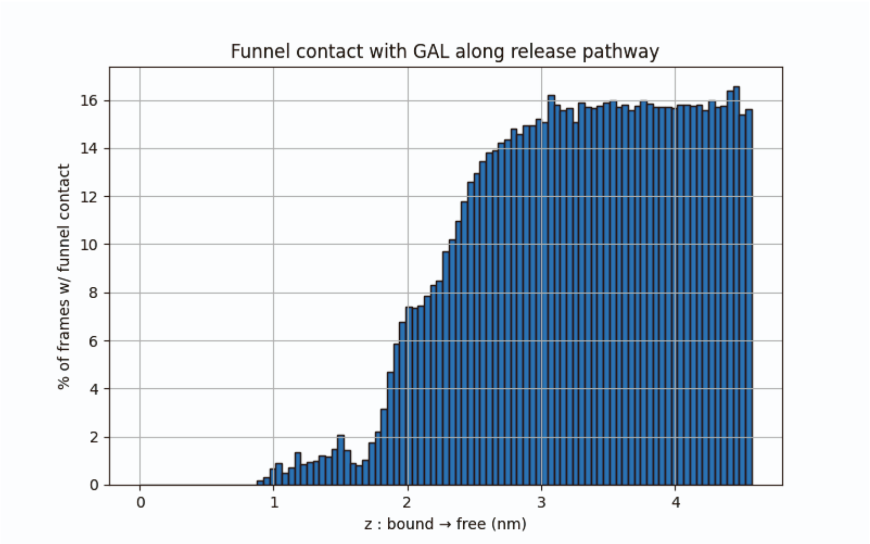

Figure S19: Interaction between GAL substrate and funnel walls along the release reaction coordinate. Bars show the percentage of frames where the restraint force from the funnel wall was not equal to 0.

Methods

MD Simulation Details. The setup of the simulations follows our previous work (1). Standard protonation states at neutral pH were assigned to all protein titratable residues (deprotonated aspartate and glutamate residues, and singly protonated histidine residues, except for H56, which forms a salt bridge with E180 and, therefore, was set as doubly protonated). The proteins were embedded in a POPC bilayer and surrounded by a 100mM NaCl solution. The overall systems were neutral. The CHARMM36m force field (3,4) was used for the protein and lipids. Ions were described using default CHARMM parameters, and the CHARMM TIP3P model (5) was used for water molecules. Protonated and deprotonated galactonate (GAL) parameters were taken from (1). They were obtained using the SwissParam server (6) in both protonated and deprotonated states. Van der Waals interactions were calculated with the Lennard–Jones potential and a cutoff radius of 1.2 nm, with forces smoothly switched to zero in the range of 1.0–1.2 nm. Electrostatic interactions were calculated by the particle mesh Ewald method (7), with a real-space cutoff distance of 1.2 nm. An integration time step of 2 fs was used. In all simulations the temperature was maintained at 310.15 K using the v-rescale thermostat (8) with a time constant of 0.5 ps. The thermostat was applied separately to the protein, lipid bilayer, and aqueous solution containing ions. The same groups were used for the removal of the center-of-mass linear motion. The systems were first equilibrated following the protocol described in (1). After that, 500 ns-long production MD runs for each of the two galactonate protomers were performed in the isothermal-isobaric ensemble using a semi-isotropic Parrinello–Rahman (9) barostat with a time constant of 0.5 ps, at the target pressure to 1 bar. The last configuration obtained from these production runs were used as the starting points for subsequent ~10 μs-long well-tempered funnel metadynamics simulations.

Well-Tempered Funnel Metadynamics Simulations. Funnel metadynamics was used to drive the galactonate unbinding process. The release coordinate (z) – also describing the axis of the funnel, was defined as the vector pointing from the center of mass of the protein through the mouth of the gating helices (Fig. S16-S17). We restrained the center-of-mass of GAL within a funnel in the shape defined by the sigmoid function

$$r(z) = R_w + \frac{R_t - R_w}{1+\exp[-4m(z-C)/(R_w-R_t)]}$$

which decays smoothly from a wide radius (R_w) to a thin radius (R_t). The inflection point (C) of the curve defines the midpoint of this decay, while the slope (m) sets the steepness of the drop-off. We used C=1.5 nm, m=1 nm, R_w=1.2 nm, and R_w=1 Å. The latter was chosen following (2). The resulting funnel restraint volume extends from 0.5 nm above the protein center-of-mass to 4.25 nm beyond and into the solvent.

The gate, which was restrained in the open conformation during metadynamics, was defined as the center of mass distance between door-1 (spanning residues 139-158) and door-2 (spanning residues 373-392). A lower bound on this distance was enforced at 1.7 nm, at which point a harmonic restraint with a hard energy constant of 25,000 kJ mol^{-1} would kick in. To stop the secondary structure in the doors from warping under the gate restraint force, a dihedral restraint

with a force constant of 1,000 kJ mol^{-1} nm^{-1} was applied to the backbone atoms of the residues in the doors.

Well-tempered metadynamics simulations (10) were performed using the PLUMED library version 2.6.2 (11) plugged into GROMACS version 2024.3 (12). Gaussian potentials with a height of 1.2 kJ/mol and a width (σ) of 0.1 nm were deposited every 500 simulation steps. A bias factor of 25 was applied to modulate the bias deposition and ensure convergence of the free-energy surface.

In order to affirm the reliability of our free energy estimates, it was necessary to mark regions of the reaction coordinate (*z*) as valid or invalid (shaded gray in Fig. 3 of the main text) and ensure that any numerical claims pointed to valid regions of the free energy surface. Namely, a region of the free energy surface was deemed invalid if GAL was feeling a restraint force from the funnel walls in >1% of frames, while interacting with DgoT in >1% of frames. In the protein bound region from z = (-0.25 to 1.2) nm, GAL was interacting with DgoT but not experiencing any bias from the funnel restraint (see Fig. S18-S19). In the transition region from z=(1.2 to 3.9) nm where the funnel narrows, GAL frequently interacts with DgoT, but the restraint force from the funnel walls biases its interactions with the protein, clouding the reliability of the surface (see Fig. S18-S19). Finally, in the solvated state z=(3.9 - 4.2) nm, the ligand is beyond the 12 Å interaction cutoff distance and is thus confirmed to experience no force from the protein (Fig. S18-S19). At this point, GAL is restrained to a homogeneous volume outside the protein, allowing for a proper volume correction to be applied, and the free energy of release to be determined, as the difference between bound and unbound states.

Release of Galactonate from Unbiased MD Simulations. We focused on the state that features deprotonated D46 (or D46[-]) and protonated E133 (E133[H]), as deprotonation of D46 was found to be crucial for substrate release in our previous classical MD simulations (1). For GAL, two protomers were considered, protonated (*p* hereafter) and deprotonated (*d*) (see Fig. 1c of the main text). The starting structures of the corresponding DgoT/GAL complexes in their inward-facing state were taken from the trajectories obtained in our previous work (1) (denoted hereafter as *root-p* and *root-d*, respectively for protonated and deprotonated GAL). Specifically, from the *root-p* simulation*,* we selected one snapshot in which the gate was opened (defined if the F137/W373 distance is greater than 8 Å) to obtain the protonated GAL and gate opened state (*p-open* system). Next, we deprotonated the GAL molecule from the latter (and remove one chloride ion to maintain neutrality). The resulting system was optimized using a steepest descent algorithm with a maximum force tolerance of 10 kJ mol^{-1} nm^{-1} on each atom. This led to the *d-open* system, with deprotonated GAL and the gate open. Complementarily, we selected a snapshot from the *root-d* simulation with the gate closed (defined if the F137/W373 distance is lower than 8 Å) to obtain the *d-closed* system. From this configuration, we protonated the GAL (and removed one sodium ion) to obtain the *p-closed* system, which was then minimized using the same protocol as for the *d-open* system. Therefore, we generated a total of four initial structures, controlling for both the protonation state of GAL and the open/closed state of the gate. These were used as starting structures for unbiased MD simulations. Specifically, for each of the four distinct states (*d-open, d-closed, p-open, p-close*) we performed four distinct 500 ns NPT runs (i.e., 16 simulations or 8 µs in total), where the initial velocities were extracted from a Maxwell-Boltzmann distribution at the target temperature of 310.15 K. These simulations were used to investigate the time for release of the substrate. This was implemented by monitoring the distance from the center of mass of GAL to that of the protein projected onto the intracellular-bound axis perpendicular to the bilayer. The simulation was stopped if this metric exceeded the z=3 nm mark, corresponding to the cytosol-membrane interface (Fig. S10). This procedure yielded the estimates of the residence time listed in Fig. S7.

Validation of the Open Gate Restraint:

The results of the unbiased MD simulations also provide important validation for our choice of restraining the intracellular gate open during the metadynamics simulations. The gate remained open in trajectories initiated with an open conformation and those started from closed conformations showed a closed gate for 500 ns (Figs. S7-S9). This indicates that the gate conformation is stable on this timescale and that its open and closed states are separated by an energy barrier larger than $k_B T$. Spontaneous substrate release was observed only in unbiased MD simulations initiated from the open-gate conformation, whereas no release occurred when the gate was closed, suggesting that gate opening is a prerequisite for release. Furthermore, no spontaneous gate closure was observed following substrate release in the 500 ns timescale of the unbiased MD simulations. This is in line with the results of ref.(12), showing that (i) deprotonation of D46 favors the open state of the intracellular gate and (ii) subsequent release of the second proton is necessary for intracellular gate closure upon substrate dissociation.

Supporting References

1. Dmitrieva, N., Gholami, S., Alleva, C., Carloni, P., Alfonso-Prieto, M., & Fahlke, C. (2024). Transport mechanism of DgoT, a bacterial homolog of SLC17 organic anion transporters. The EMBO Journal, 43(24), 6740–6765. https://doi.org/10.1038/s44318-024-00279-y
2. Raniolo, S., & Limongelli, V. (2020). Ligand binding free-energy calculations with funnel metadynamics. Nature Protocols, 15(9), 2837–2866. https://doi.org/10.1038/s41596-020-0342-4
3. Klauda, J. B., Venable, R. M., Freites, J. A., O'Connor, J. W., Tobias, D. J., Mondragon-Ramirez, C., Vorobyov, I., MacKerell, A. D., & Pastor, R. W. (2010). Update of the CHARMM All-Atom Additive Force Field for Lipids: Validation on Six Lipid Types. The Journal of Physical Chemistry B, 114(23), 7830–7843. https://doi.org/10.1021/jp101759q
4. Huang, J., Rauscher, S., Nawrocki, G., Ran, T., Feig, M., de Groot, B. L., Grubmüller, H., & MacKerell, A. D. (2016). CHARMM36m: an improved force field for folded and intrinsically disordered proteins. Nature Methods, 14(1), 71–73. https://doi.org/10.1038/nmeth.4067
5. Jorgensen, W. L., Chandrasekhar, J., Madura, J. D., Impey, R. W., & Klein, M. L. (1983). Comparison of simple potential functions for simulating liquid water. The Journal of Chemical Physics, 79(2), 926–935. https://doi.org/10.1063/1.445869
6. Zoete, V., Cuendet, M. A., Grosdidier, A., & Michielin, O. (2011). SwissParam: A fast force field generation tool for small organic molecules. Journal of Computational Chemistry, 32(11), 2359–2368. Portico. https://doi.org/10.1002/jcc.21816
7. Essmann, U., Perera, L., Berkowitz, M. L., Darden, T., Lee, H., & Pedersen, L. G. (1995). A smooth particle mesh Ewald method. The Journal of Chemical Physics, 103(19), 8577–8593. https://doi.org/10.1063/1.470117
8. Bussi, G., Donadio, D., & Parrinello, M. (2007). Canonical sampling through velocity rescaling. The Journal of Chemical Physics, 126(1). https://doi.org/10.1063/1.2408420
9. Parrinello, M., & Rahman, A. (1981). Polymorphic transitions in single crystals: A new molecular dynamics method. Journal of Applied Physics, 52(12), 7182–7190. https://doi.org/10.1063/1.328693
10. Barducci, A., Bussi, G., & Parrinello, M. (2008). Well-Tempered Metadynamics: A Smoothly Converging and Tunable Free-Energy Method. Physical Review Letters, 100(2). https://doi.org/10.1103/physrevlett.100.020603
11. Tribello, G. A., Bonomi, M., Branduardi, D., Camilloni, C., & Bussi, G. (2014). PLUMED 2: New feathers for an old bird. Computer Physics Communications, 185(2), 604–613. https://doi.org/10.1016/j.cpc.2013.09.018
12. Abraham, M. J., Murtola, T., Schulz, R., Páll, S., Smith, J. C., Hess, B., & Lindahl, E. (2015). GROMACS: High performance molecular simulations through multi-level parallelism from laptops to supercomputers. SoftwareX, 1–2, 19–25. https://doi.org/10.1016/j.softx.2015.06.001
